# Anticancer Activity of Erianin: Cancer-Specific Target Prediction Based on Network Pharmacology

**DOI:** 10.3389/fmolb.2022.862932

**Published:** 2022-03-17

**Authors:** Lili Yan, Zhen Zhang, Yanfen Liu, Shuyi Ren, Zhiyu Zhu, Lu Wei, Jiao Feng, Ting Duan, Xueni Sun, Tian Xie, Xinbing Sui

**Affiliations:** ^1^ School of Pharmacy, Hangzhou Normal University, Hangzhou, China; ^2^ Key Laboratory of Elemene Class Anti-Cancer Chinese Medicines, Engineering Laboratory of Development and Application of Traditional Chinese Medicines, Collaborative Innovation Center of Traditional Chinese Medicines of Zhejiang Province, Hangzhou Normal University, Hangzhou, China; ^3^ Department of Orthopedic Surgery, Hangzhou Orthopedic Institute, Affiliated Hangzhou First People’s Hospital, Zhejiang University School of Medicine, Hangzhou, China

**Keywords:** erianin, anticancer activity, target prediction, molecular docking, ADMET

## Abstract

Erianin is a major bisbenzyl compound extracted from *Dendrobium chrysotoxum* Lindl., an important traditional Chinese herb. In recent years, a growing body of evidence has proved the potential therapeutic effects of erianin on various cancers, including hepatoma, melanoma, non-small-cell lung carcinoma, myelogenous leukemia, breast cancer, and osteosarcoma. Especially, the pharmacological activities of erianin, such as antioxidant and anticancer activity, have been frequently demonstrated by plenty of studies. In this study, we firstly conducted a systematic review on reported anticancer activity of erianin. All updated valuable information regarding the underlying action mechanisms of erianin in specific cancer was recorded and summarized in this paper. Most importantly, based on the molecular structure of erianin, its potential molecular targets were analyzed and predicted by means of the SwissTargetPrediction online server (http://www.swisstargetprediction.ch). In the meantime, the potential therapeutic targets of 10 types of cancers in which erianin has been proved to have anticancer effects were also predicted *via* the Online Mendelian Inheritance in Man (OMIM) database (http://www.ncbi.nlm.nih.gov/omim). The overlapping targets may serve as valuable target candidates through which erianin exerts its anticancer activity. The clinical value of those targets was subsequently evaluated by analyzing their prognostic role in specific cancer using Kaplan-Meier plotter (http://Kmplot.com/analysis/) and Gene Expression Profiling Interactive Analysis (GEPIA) (http://gepia.cancer-pku.cn/). To better assess and verify the binding ability of erianin with its potential targets, molecular flexible docking was performed using Discovery Studio (DS). The valuable targets obtained from the above analysis and verification were further mapped to the Kyoto Encyclopedia of Genes and Genomes (KEGG) pathway using the Database for Annotation, Visualization and Integrated Discovery (DAVID) (http://david.abcc.ncifcrf.gov/) to explore the possible signaling pathways disturbed/regulated by erianin. Furthermore, the *in silico* prediction of absorption, distribution, metabolism, excretion, and toxicity (ADMET) properties of erianin was also performed and provided in this paper. Overall, in this study, we aimed at 1) collecting all experiment-based important information regarding the anticancer effect and pharmacological mechanism of erianin, 2) providing the predicted therapeutic targets and signaling pathways that erianin might act on in cancers, and 3) especially providing *in silico* ADMET properties of erianin.

## 1 Introduction

**FIGURE 1:**
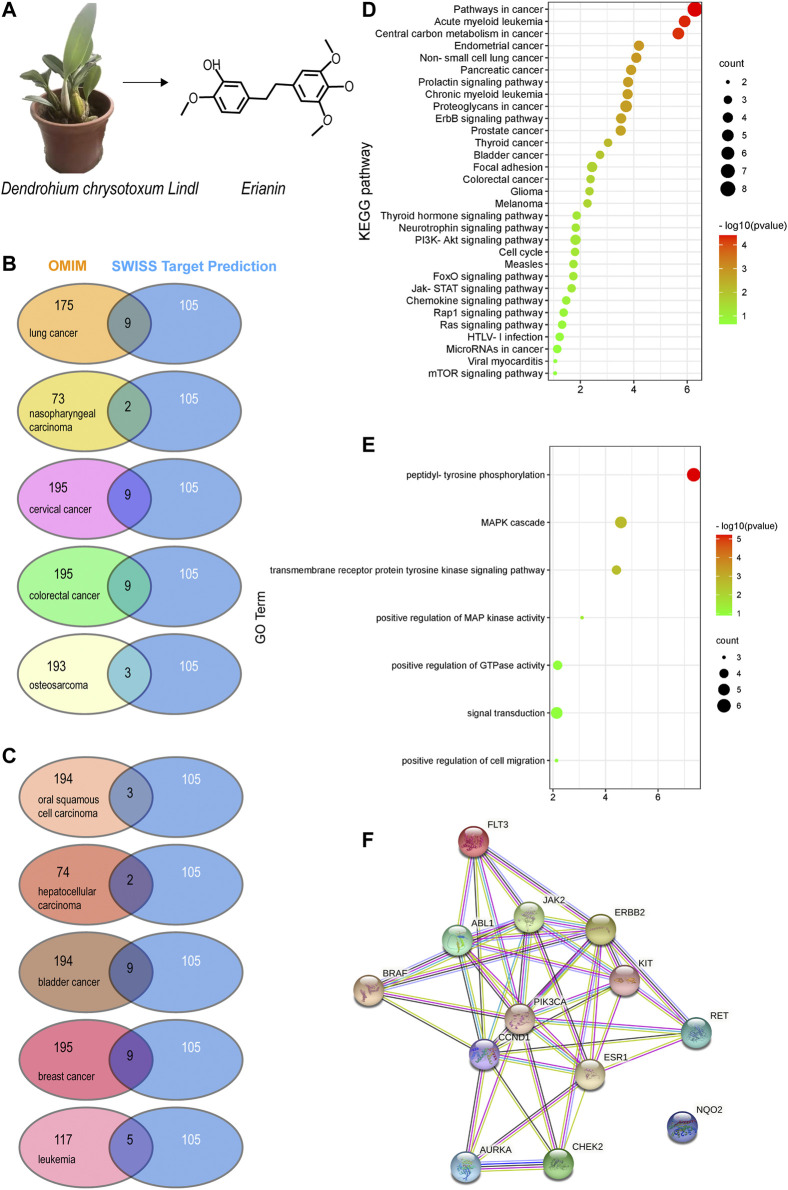
Potential target prediction and analysis of the KEGG pathway, GO_BP, and target–target interaction. **(A)** Molecular structure of erianin; **(B–C)** overlapping target analysis by Venn diagrams; **(D)** KEGG pathway enrichment of the overlapping targets; **(E)** GO_biological process analysis of the overlapping targets; **(F)** target–target interaction of the overlapping targets analyzed by STRING.

*Dendrobium chrysotoxum* Lindl. is a traditional Chinese medicine with a wide range of clinical applications since as early as the 28th century BC because of its tonic, astringent, analgesic, and anti-inflammatory properties (Wang, 2021). Erianin is a bibenzyl compound extracted from *Dendrobium chrysotoxum* (Figure 1A). It has been included in the Pharmacopoeia of the People’s Republic of China (2020 version) and considered a representative index component for the quality control of *Dendrobium chrysotoxum* (Yang et al., 2021). Liu et al. (2019b) recently built a miniature mass method for the determination of phenol components in *Dendrobium chrysotoxum*, which revealed that the content of bibenzyl compounds was the highest in this herb*.* Among these bibenzyl compounds, erianin was determined as the typical chemical marker from *Dendrobium chrysotoxum* (Xie et al., 2021). Modern pharmacological studies have shown that erianin can exert therapeutic activities under various disease conditions such as tumors (Liu et al., 2019a; Chen et al., 2020a; Dong et al., 2020), inflammation (Zhang et al., 2019a; Zhang et al., 2019b; Dou et al., 2020; Yang et al., 2021), and oxidative injury (Chen et al., 2019). In this study, we focus on the anti-tumor activities of erianin and its underlying mechanism of action. In general, erianin exerts its anticancer activities by inhibiting tumor migration, invasion, and angiogenesis and inducing inflammation, apoptosis, autophagy, and G2/M cycle arrest as reported previously in a variety of cancers. All of the related publications were deeply mined in PubMed using “erianin” as the keyword and systematically reviewed and summarized in this paper. Most importantly, valuable information on erianin about its potential targets and their clinical value were analyzed and provided in this paper. Last but not least, the *in silico* prediction of ADMET properties of erianin was also performed and included in this study.

## 2 Materials and Methods

### 2.1 Data Retrieval

Literature mining was carried out in PubMed (https://pubmed.ncbi.nlm.nih.gov) with “erianin” as the only keyword. Restrictions such as article type, publication date, language, and journal were not set during data retrieval in order to collect all relevant articles as thoroughly as possible. As a result, 52 publications were extensively identified and carefully reviewed. Those identified literature studies include studies of mass spectrometry–based method development for erianin determination from complex matrix, and the anti-inflammatory, antioxidative, and anticancer activity evaluation of erianin with various *in vitro* and *in vivo* models, of which nearly half of the identified literature studies are related to the evaluation of its anticancer activity and collected in this study. The valuable information regarding the action mechanisms of erianin such as its targeting/regulating molecules and pathways in those related literature studies was then recorded and summarized.

### 2.2 Potential Target Prediction of Erianin

SwissTargetPrediction (http://www.wisstargetprediction.ch) is a common database for predicting compound targets and used to predict the potential targets of erianin. OMIM (http://www.omim.org/) is a comprehensive database of human genes and genetic diseases and used to predict potential therapeutic targets for specific cancer. Discovery Studio 2020 (DS) is a highly visual commercial software integrated by BIOVIA for life science research and used to perform flexible docking to investigate the binding affinity of erianin to corresponding targets. DS receptors and ligands were obtained from the PDB database (https://www.rcsb.org/) and PubChem database (https://pubchem.ncbi.nlm.nih.gov/), respectively. The PDB numbers of the receptor proteins are RET:6NJA, FLT3:1RJB, c-KIT:6HH1, PI3K:3LI3, and ABL1:4WA9. It was then processed as follows: Ligand preparation: tools–Small molecule–Prepare or filter ligands–Prepare ligands–run; Simulation–Change forcefield–apply forcefield. Receptor preparation: 1. Water–delete; 2. Macromolecules–prepare protein–clean protein; 3. Chemistry–Hydrogens; 4. Define and Edit Binding site–Define receptor; 5. Receptor–Ligand Interaction–Define site–From receptor cavities; 6. Change forcefield–Apply forcefield. Parameters such as CDOCKER Energy and ChiFlex Energy were used to evaluate the molecular docking results. The conformation with the highest CDOCKER energy score value was selected as the binding conformation for further analysis.

### 2.3 Analysis of the Prognostic Role of Predicted Targets in Specific Cancer

KM-plotter (http://kmplot.com/analysis/) and GEPIA (http://gepia.cancer-pku.cn/) are online tools used to assess the correlation of predicted targets with survival in specific cancer in this study. With the help of the above two tools, the overall survival (OS) of generalized cancer was analyzed according to gene symbols with the log-rank test, and the prognostic results were then screened out.

### 2.4 Absorption, Distribution, Metabolism, Excretion, and Toxicity Prediction

The prediction of ADMET properties of erianin including aqueous solubility, blood–brain barrier penetration, human intestinal absorption (HIA), hepatotoxicity, cytochrome P450 2D6 inhibition, and plasma protein binding was calculated and computed using ADMET Descriptors and Filter by Lipinski tools available with Discovery Studio 2020. The primary steps include Small Molecules–Calculate Molecular Properties–ADMET Descriptors–pk-test (all)–run. Toxicity analysis of erianin was conducted using the “TOPKAT” module in Discovery Studio. Toxicity parameters including rodent carcinogenicity, mutagenicity, aerobic biodegradability, and rat oral LD50 were recorded.

## 3 Results and Discussion

### 3.1 Literature Review: Erianin Showed Potential Anticancer Activity *via* Different Modes of Action in a Wide Range of Cancers

#### 3.1.1 Erianin Exerts Anticancer Activity by Anti-Angiogenesis and Inhibiting Cancer Cell Proliferation, Invasion, and Migration

Erianin belongs to the Cobutin class and can competitively bind to tubulin due to a colchicine-like structure. It was found that erianin can inhibit retinal angiogenesis in retinal endothelial and microglial cells (Yu et al., 2016). Further investigation found that the inhibition of erianin on retinal neoangiogenesis was attributed to its role in abrogating high-glucose–induced VEGF (vascular endothelial growth factor) expression *via* blocking ERK1/2-mediated HIF-1α (hypoxia-inducible factor-1α). Besides, erianin can cause endothelium-specific metabolism disturbance, further suggesting its potential effects on anti-angiogenesis (Gong et al., 2004a). Tumor angiogenesis is a key process in tumor growth and metastasis. In recent years, erianin was found to be a promising anticancer drug, partially due to its anti-angiogenesis activity. It was reported that erianin can induce endothelial cytoskeletal disorganization by depolymerizing both F-actin and beta-tubulin, thereby inhibiting angiogenesis and cell growth in hepatoma and melanoma *in vitro* and *in vivo* models (Gong et al., 2004b). The anti-angiogenesis activity of erianin was also reported in 2LL cells (Su et al., 2017). In that study, erianin was found to exert anti-angiogenesis activity *via* suppressing the expression and activity of indoleamine 2,3-dioxygenase (IDO), an enzyme which plays a role in regulating tumor angiogenesis in addition to its immune function, therefore inhibiting IDO-induced metastasis and invasion in 2LL cells. Programmed cell death ligand 1 (PD-L1) is another factor confirmed to play a vital role in tumor angiogenesis, mainly through its regulation in VEGF expression. The inhibitory activity of erianin on PD-L1–mediated angiogenesis was also demonstrated in cervical cancer (Yang et al., 2021). HeLa cells treated with erianin showed significant inhibition on PD-L1–mediated upregulation of VEGF. Silencing of PD-L1 reduced tube formation which can be increased by erianin treatment, further confirming the anti-angiogenic property of erianin. In addition, erianin can exert anticancer activity by inhibiting cell proliferation, migration, and invasion. Regulations of PI3K/AKT and ERK/P38 signaling pathways were reported to be involved in erianin-induced inhibition on cell proliferation, migration, and invasion in liver cancer cells (Yang et al., 2020a). Cells treated with erianin can significantly decrease the phosphorylation of AKT (protein kinase B), ERK (extracellular signal–regulated kinase), and P38. More recently, erianin was found to be able to inhibit cell growth and migration in non-small-cell lung cancer and trigger ferroptosis in a calcium/calmodulin-dependent manner (Chen et al., 2020a). Ferroptosis is a newly defined form of regulated cell death which has attracted great interest in the field of cancer research. In a recent study, erianin was reported to induce ferroptosis in bladder cancer cells *via* NRF2 (nuclear factor erythroid 2–related factor 2) inactivation (Xiang et al., 2021).

#### 3.1.2 Erianin Exerts Anticancer Activity by Inducing Apoptosis

Induction and activation of apoptosis and related cell death networks play an important role in cancer therapies. Cells undergo apoptosis mainly through three pathways, namely, the death receptor–dependent extrinsic pathway, the mitochondria-mediated intrinsic pathway, and the intrinsic endoplasmic reticulum (ER) pathway. The extrinsic death receptor pathway is triggered by the binding of death ligands to a death receptor, contributing to the formation of the death-inducing signaling complex (DISC) and subsequently leading to the activation of caspase-8 (Zhang et al., 2019b). The intrinsic mitochondrial pathway is initiated within the cell. It can be triggered by stimuli such as irreparable genetic damage, hypoxia, extremely high concentrations of cytosolic Ca^2+^, and severe oxidative stress. The mitochondria-mediated intrinsic pathway increases the mitochondrial permeability and the release of pro-apoptotic molecules such as cytochrome-c into the cytoplasm and subsequently leads to the activation of cysteinyl aspartate–specific proteinase (caspase) (Zhang et al., 2019b). The intrinsic endoplasmic reticulum pathway was thought to be a caspase 12–dependent and mitochondria-independent apoptotic pathway; however, it is much less well known compared to the death receptor–dependent pathway and mitochondria-mediated pathway (Wong, 2011). Both of the intrinsic and extrinsic pathways have been extensively studied in terms of the anti-tumor effects of erianin. It was reported to be able to induce apoptosis in human nasopharyngeal carcinoma (NPC) cells *via* mitochondrial and Fas-mediated pathways (Liu et al., 2019b). NPC cells treated with erianin exhibited a significant increase in the activation of death receptors and caspase cascades (caspase-3, -8, -9), as well as alteration in mitochondrial membrane potentials, all of which are directly related to cell apoptotic pathways. In cervical cancer cells, erianin was found to induce mitochondrial-based apoptosis *via* regulating the expression of ERK and tumor suppressor gene p53, possibly due to its regulating role in the expression and activation of apoptosis-associated Bcl-2 (B-cell leukemia/lymphoma 2) and Bax (Bcl-2–associated X protein) (Li et al., 2018). Bcl-2 is an anti-apoptotic protein. One study conducted with T47D cells demonstrated that erianin can induce cell apoptosis through reducing the expression of Bcl-2 and activating caspase signaling (caspase-3, -7, -9) (Sun et al., 2016). In addition, oxidative-stress–mediated apoptosis was also reported to be involved in the anticancer process of erianin. Using HepG2 and SMMC-7721 cells *in vitro* and xenograft models, Zhang et al. (2019a) have investigated the anti-carcinogenesis property of erianin in liver cancer. It was found that treatment with erianin in liver cancer cells increased the intracellular levels of reactive oxygen species and the expression of pro-apoptosis proteins and reduced the cell mitochondrial membrane potential and the expression of anti-apoptosis proteins. It turned out that the anti-liver cancer property of erianin was highly correlated to its modulation of oxidative-stress–mediated mitochondrial apoptosis (Zhang et al., 2019a). The induction of mitochondrial apoptosis by erianin was also confirmed in bladder cancer cells as is reported by Zhu et al. (2019). Moreover, in osteosarcoma cells, erianin was found to be able to activate both the extrinsic and intrinsic apoptotic pathways as demonstrated by mitochondrial depolarization, the activation of caspase-3, -8, -9, and PARP (poly ADP-ribose polymerase), the decreased expression of Bcl-2 and Bcl-xl (B-cell lymphoma-extra-large), and surviving in erianin-treated osteosarcoma cells (Wang et al., 2016). Caspase-dependent apoptosis induced by erianin was also confirmed in oral squamous cell carcinoma cells (Chen et al., 2020b). It appears that induction of apoptosis through either the extrinsic or the intrinsic pathway is a common way for erianin to exert anticancer activity in different types of cancers.

#### 3.1.3 Erianin Exerts Anticancer Activity by Inducing Cell Cycle Arrest

Many studies have confirmed that erianin can promote cell cycle arrest, mainly in the G2/M phase. This phenomenon has been frequently reported in different types of cancers such as hepatocellular carcinoma (Dong et al., 2020), lung cancer (Chen et al., 2020a), colorectal cancer (Sun et al., 2020), breast cancer (Sun et al., 2016), bladder cancer (Zhu et al., 2019), and osteosarcoma (Wang et al., 2016). However, the underlying molecular mechanism is not fully studied. The vast majority of the studies analyzed cell cycle distribution before and after erianin treatment in specific cancer cells and assessed erianin-induced alteration of cell cycle–related gene expression by q-PCR or western blotting. Those studies suggest that erianin may promote cell cycle arrest mainly *via* modulating the expression of CDKs (cyclin-dependent kinases), cyclin B1, CDC25C (cell division cycle 25C), p21, and p27. Recently, Dong et al. (2020) carried out a transcriptome sequencing analysis in hepatocellular carcinoma cells with/without erianin treatment, and followed by GSEA (gene set enrichment analysis). The result of core gene enrichment suggests that erianin may induce G2/M cell cycle arrest in hepatocellular carcinoma cells mainly through regulating G2/M checkpoints. Further protein level analysis showed elevated expressions of PLK1 (polo-like kinase 1), Aurora A, and cyclin B1 in cells treated with erianin. Moreover, they also found that erianin can induce DNA damage in hepatocellular carcinoma cells. The intensity of pH2AX (phospho-histone 2AX), a DNA damage mark, was significantly increased in erianin-treated HCC cells.

#### 3.1.4 Erianin Exerts Anticancer Activity Through Regulating Immune Inflammatory Response

Immune modulation has become an important research topic in cancer therapy. Growing evidence suggests that various immune cells contribute to tumor progression when present in the tumor microenvironment (TME). A study has found that erianin can reduce immune inflammatory responses in dextran sulfate sodium–induced ulcerative colitis, possibly *via* activating TLR4 (Toll-like receptor 4) and STAT3 (signal transducer and activator of transcription 3) signaling pathways (Dou et al., 2020). Recently, researchers found that erianin can modulate immune response to exert its anti-carcinogenesis property in liver cancer cells (Zhang et al., 2019a). They examined the levels of 111 types of cytokines in HepG2-xenografted and SMMC-7721–xenografted tumor tissues. The results showed that 38 and 15 types of cytokines in HepG2-xenografted and SMMC-7721–xenografted tumors, respectively, were substantially influenced by erianin treatment, most of which are involved in immune functions. Although the results obtained from cytokine detection pointed to the modulation of T cell functions induced by erianin in liver cancer, it somewhat lacks direct evidence to confirm it. More recently, Yang et al. (2021) conducted a study using a co-cultured HeLa and T cell model and confirmed that erianin can restore tumor-killing activity of T-lymphocytes and significantly enhance the specific cleavage of HeLa cells by T cells. Downregulation of PD-L1 expression and upregulation of its lysosomal degradation were found to be the vital mechanisms involved. The decreased expression of PD-L1 on the surface of tumor cells reduced its interaction with T cells *via* PD-1 which is expressed on the surface of T cells, thereby improving the killing effect of T cells. However, how erianin affects the expression of PD-L1 is not fully elucidated. Besides, erianin has recently been identified as an inhibitor of NLRP3 (NOD-like receptor family pyrin domain containing 3) (Zhang et al., 2021b). The NLRP3 inflammasome can be widely found in immune cells and play important roles in a wide variety of inflammatory diseases. Pharmacologically targeting NLRP3 has been considered a promising strategy to treat NLRP3-related inflammatory diseases (Mangan et al., 2018). Erianin was found to be able to directly interact with NLRP3, therefore inhibiting the NLRP3 inflammasome assembly (Zhang et al., 2021b). This further supports the potential roles of erianin in modulating inflammatory responses in cancer therapy.

#### 3.1.5 Other Signaling Pathways Involved in Anticancer Mechanisms of Erianin

In addition to the anti-angiogenesis activity, induction of apoptosis and cell cycle arrest, and modulation of immune inflammatory response, some other signaling pathways were also involved in the anticancer action of erianin. As is reported by Zhang et al. (2021a), erianin can inhibit the growth of human lung cancer cells by affecting the PI3K/AKT/mTOR signaling pathway. In their study, they firstly explored the possible targets of erianin by molecular docking and then verified those targets *via* western blotting. They have found that erianin can effectively downregulate the phosphorylation of PI3K, AKT, and mTOR (mammalian target of rapamycin), all of which were also proved to show high binding affinity to erianin by molecular docking. The MAPK (mitogen-activated protein kinase) signaling pathway is closely related to cell proliferation, migration, apoptosis, and autophagy. Continuous activation of the MAPK signaling pathway is frequently found in varieties of malignant tumors. According to a recently published study conducted in oral squamous cell carcinoma cells, erianin can induce both apoptosis and autophagy by regulating the MAPK signaling pathway, which has extensive cross talk with the PI3K/AKT/mTOR pathway (Chen et al., 2020b). Besides, erianin was found to be able to suppress hepatocellular carcinoma cells through downregulating PI3K/AKT and MAPK signaling pathways (Yang et al., 2020a) and inhibit gastric cancer cell growth through suppressing the HRAS–PI3K–AKT signaling pathway (Wang et al., 2021). In fact, double targeting MAPK and PI3K cascades has been considered a rational combined therapeutic strategy in cancer therapy due to its vital role in regulating physiological processes and the limitation of single pathway inhibitors in clinical success (Ciuffreda et al., 2014).

#### 3.1.6 Investigation of Combinatorial Therapy of Erianin With Other Anticancer Drugs

Synergistic drug combination has been a hot research topic due to its unique advantages in improving drug efficacy, reducing drug adverse side effects, and overcoming drug resistance. Natural products particularly possess some advantages in this concern such as multi-target and multi-channel effects and relatively low toxicity. Recently, the synergistic effect of erianin combined with doxorubicin was assessed in breast cancer cells (Goel et al., 2018). According to the results obtained from that study, mainly cytotoxicity and cell proliferation test, researchers demonstrated that the combination of erianin with doxorubicin showed synergistic anticancer effect or the stronger inhibitory effect on breast cancer cells. However, whether such synergistic effects still hold when applied to other types of cancers and what are the underlying mechanisms of action involved remain unclear. More recently, erianin was found to be able to inhibit the proliferation of oxaliplatin-resistant human colon cancer cells and suppress the cell cycle in the G2/M phase (Su et al., 2017). Inhibition of the STAT3 signaling pathway and decreased expression of P-glycoprotein (P-gp) by erianin were thought to be the mechanisms involved. However, further investigations regarding the potential application of erianin in the combinatorial therapeutic regimen are definitely necessary before its clinical application.

### 3.2 Potential Target Prediction and Evaluation

#### 3.2.1 Prediction of Potential Molecular Targets of Erianin

Based on the molecular structure of erianin (Figure 1A), 105 potential targets of erianin were predicted using the SwissTargetPrediction online tool (Daina et al., 2019) (Supplementary Table S1). According to the literature review conducted above, anticancer effects of erianin were mainly evaluated in 10 types of cancers, including lung cancer, oral squamous cell carcinoma, nasopharyngeal carcinoma, hepatocellular carcinoma, cervical cancer, bladder cancer, colorectal cancer, breast cancer, osteosarcoma, and leukemia. Potential therapeutic targets of these 10 types of cancers were thus explored and predicted using OMIM (Amberger et al., 2015) (Supplementary Tables S2, S3). The overlapping targets displayed in Supplementary Tables S1–S3 were then analyzed using Venn diagrams (Figures 1B,C; Table 1). They were considered valuable targets and subsequently submitted to further evaluation.

**TABLE 1:** A list of overlapping targets of predicted erianin targets and cancer-specific therapeutic targets obtained from Venn diagrams.

Cancer type	Overlapping targets
Lung cancer	CCND1
Cervical cancer	CHEK2
Colorectal cancer	EPHB2
Bladder cancer	BRAF
Breast cancer	ESR1
	PIK3CA
	AURKA
	ERBB2
	NQO2
Oral squamous cell carcinoma	BRAF
Osteosarcoma	PIK3CA
	KIT
Nasopharyngeal carcinoma	RET
Hepatocellular carcinoma	PIK3CA
Leukemia	ABL1
	FLT3
	JAK2
	KIT
	ERBB2

**FIGURE 2:**
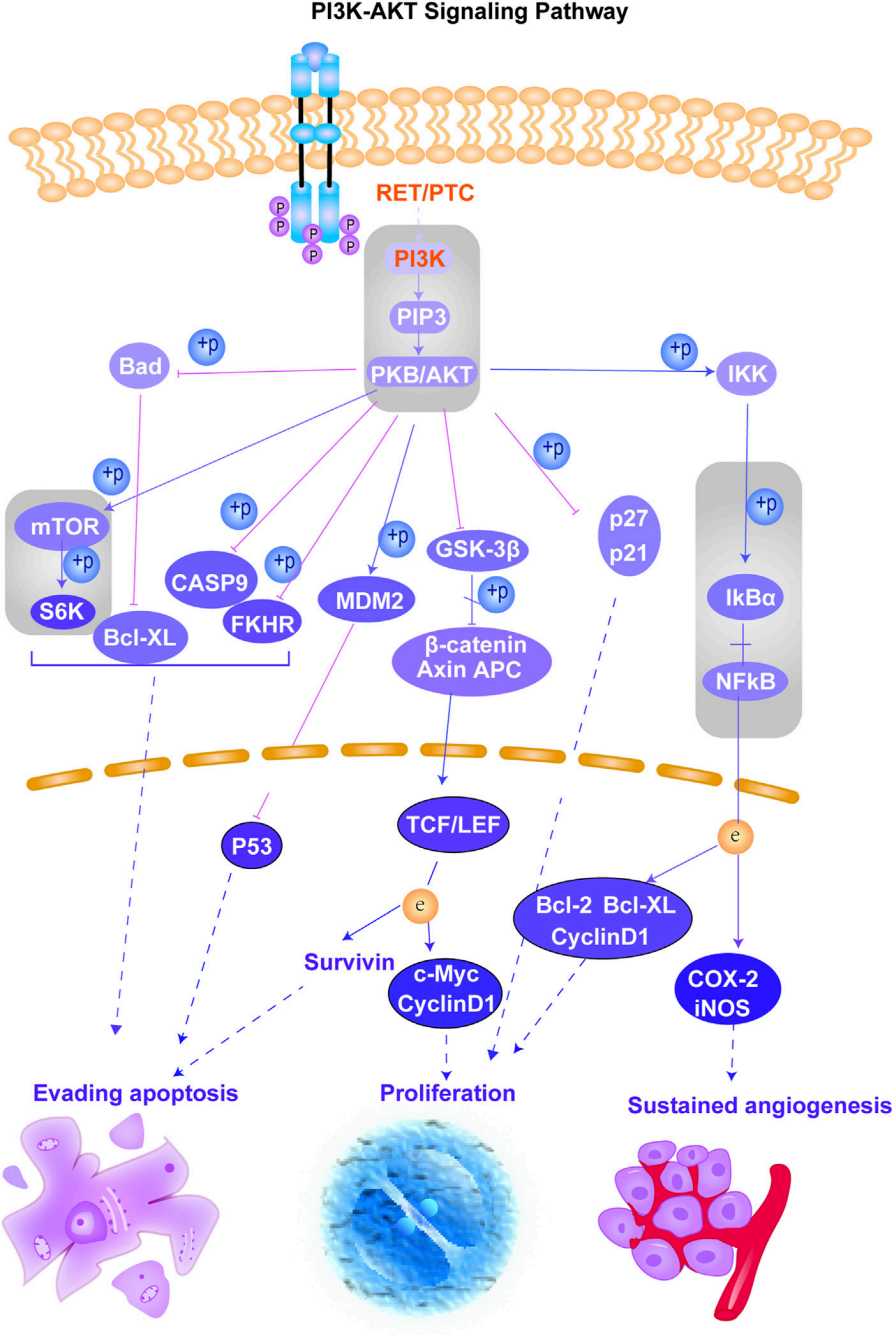
Schematic of the PI3K–AKT signaling pathway. This figure shows the important cascades associated with the PI3K–AKT signaling pathway that involves in cell angiogenesis, proliferation, and apoptosis. The proteins marked in red represent the potential targets of erianin predicted in this study. The reported signaling pathways involved in anticancer mechanisms of erianin are framed in gray.

**FIGURE 3:**
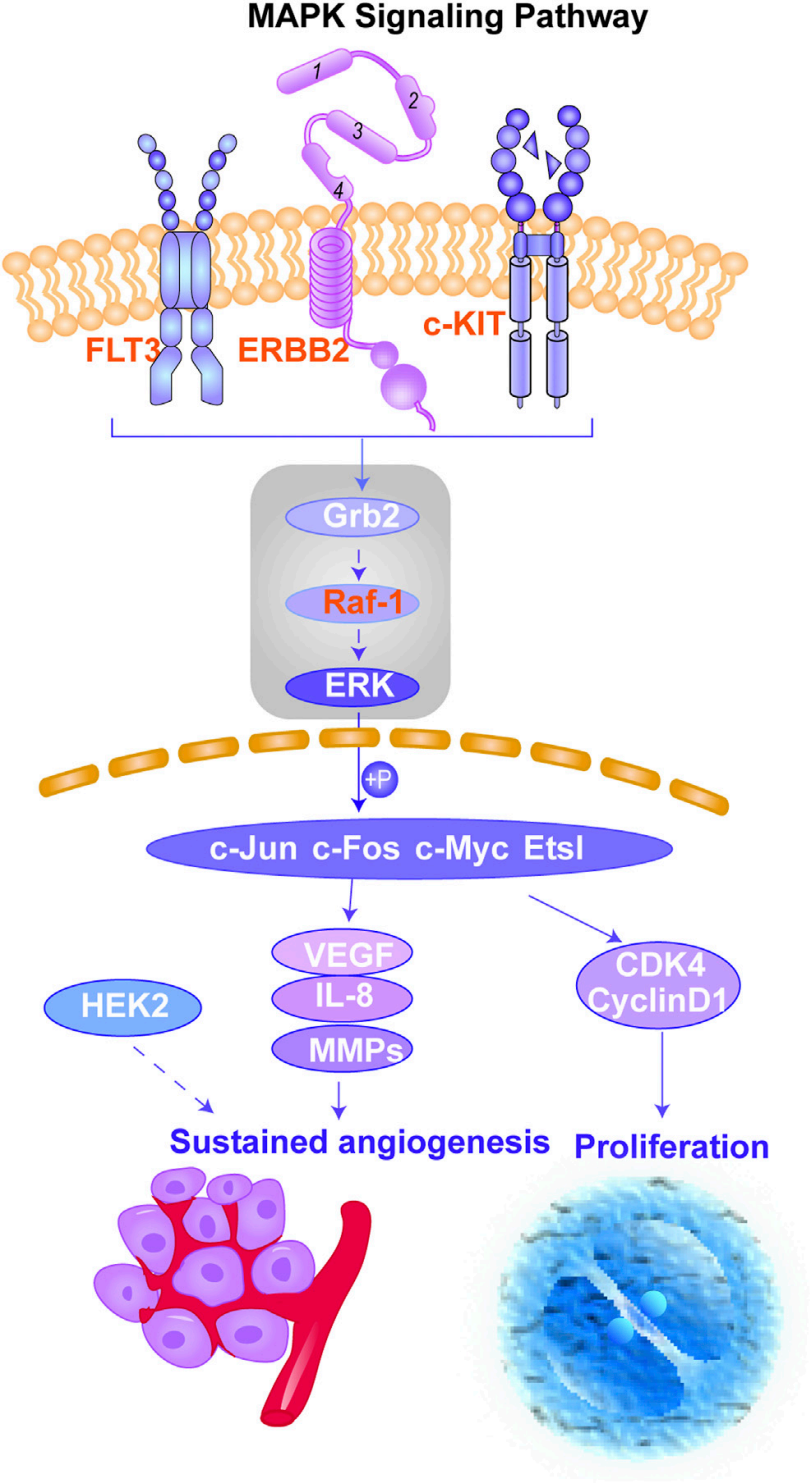
Schematic of the MAPK signaling pathway. This figure shows the important cascades associated with the MAPK signaling pathway that involves in angiogenesis and cancer cell proliferation. The proteins marked in red represent the potential targets of erianin predicted in this study. The reported signaling pathways involved in anticancer mechanisms of erianin are framed in gray.

According to the results obtained from OMIM and SwissTargetPrediction, five types of cancers (lung cancer, cervical cancer, bladder cancer, colorectal cancer, and breast cancer) share the same nine therapeutic targets when erianin is used as the therapeutic drug (Table 1). Those targets are CCND1 (cyclin D1), CHEK2 (checkpoint kinase 2), EPHB2 (EPH receptor B2), BRAF (B-Raf proto-oncogene, serine/threonine kinase), ESR1 (estrogen receptor 1), PIK3CA (phosphatidylinositol-4,5-bisphosphate 3-kinase catalytic subunit alpha), AURKA (Aurora kinase A), ERBB2 (Erb-b2 receptor tyrosine kinase 2), and NQO2 (N-ribosyldihydronicotinamide: quinone reductase 2), most of which were not previously investigated and reported. BRAF, PIK3CA, and KIT (KIT proto-oncogene, receptor tyrosine kinase) are three potential action targets of erianin in oral squamous cell carcinoma and osteosarcoma, while RET (Ret proto-oncogene, tyrosine-protein kinase receptor Ret) and PIK3CA are two targets in nasopharyngeal carcinoma and hepatocellular carcinoma that erianin may act on (Figures 1B,C; Table 1). Besides, erianin may exert anticancer activity by targeting ABL1 (ABL proto-oncogene 1, non-receptor tyrosine kinase), FLT3 (fms-related receptor tyrosine kinase 3), JAK2 (Janus kinase 2), KIT, and ERBB2 in leukemia (Figure 1C; Table 1). These targets were subsequently mapped to KEGG pathways and GO (Gene Ontology) term analysis using DAVID (https://david.ncifcrf.gov) (Dennis et al., 2003) to explore the possible signaling pathways or biological process through which erianin exerts its anticancer activity in these cancers (Figures 1D,E). Of all the targets predicted based on the erianin structure, JAK2, KIT, CCND1, PIK3CA, and BRAF are closely related to PI3K–AKT and MAPK signaling pathways (Figures 2, 3), which are broadly in line with previous studies discussed above (Chen et al., 2020b; Yang et al., 2020a). The reported cascades involved in PI3K and MAPK signaling pathways are framed in gray in Figures 2, 3, respectively. The predicted targets in this study are relatively upstream of the studied signaling pathways regulated by erianin and thus may serve as explanations, or in part, for the anticancer mechanisms of erianin. Besides, of the above targets, JAK2, PIK3CA, and BRAF are also involved in the chemokine signaling pathway, which play important roles in inflammatory immune response. Actually, erianin has been reported to be able to downregulate the phosphorylation of JAK2 and STAT3 in 2LL cells, and thus inhibit the expression of several inflammatory mediators, such as COX-2 (cyclooxygenase-2), HIF-1α, and IL-6 (interleukin-6) (Su et al., 2017). Moreover, PIK3CA, as one of most important regulatory molecules involved in PI3K–AKT and MAPK signaling pathways, has been frequently identified as a target of erianin in various cancers (Yang et al., 2020a; Wang et al., 2021; Xu et al., 2021; Zhang et al., 2021a). According to KEGG pathway analysis, KIT, ERBB2, FLT3, PIK3CA, and RET are enriched in central carbon metabolism in cancer, while ABL1, CHEK2, and CCND1 are highly related to the cell cycle process, both of which are relatively less explored. Moreover, target–target interactions were also analyzed by STRING (version 11.5) (https://cn.string-db.org/), which indicates high interactions of targets between each other, except NQO2 (Figure 1F). NQO2 is a flavoprotein that catalyzes the two-electron reduction of various quinones, redox dyes, and the vitamin K menadione. It has been frequently identified as a promising therapeutic target in addition to NQO1 [NAD(P)H: quinone oxidoreductase 1] in cancers due to its vital roles played in cancer initiation and progression, possibly *via* regulating the generation of ROS (Hussein et al., 2019; Zhang et al., 2020; Schepers et al., 2021). NQO2 might serve as a valuable target for erianin and be worth future investigation.

#### 3.2.2 Molecular Docking

**FIGURE 4:**
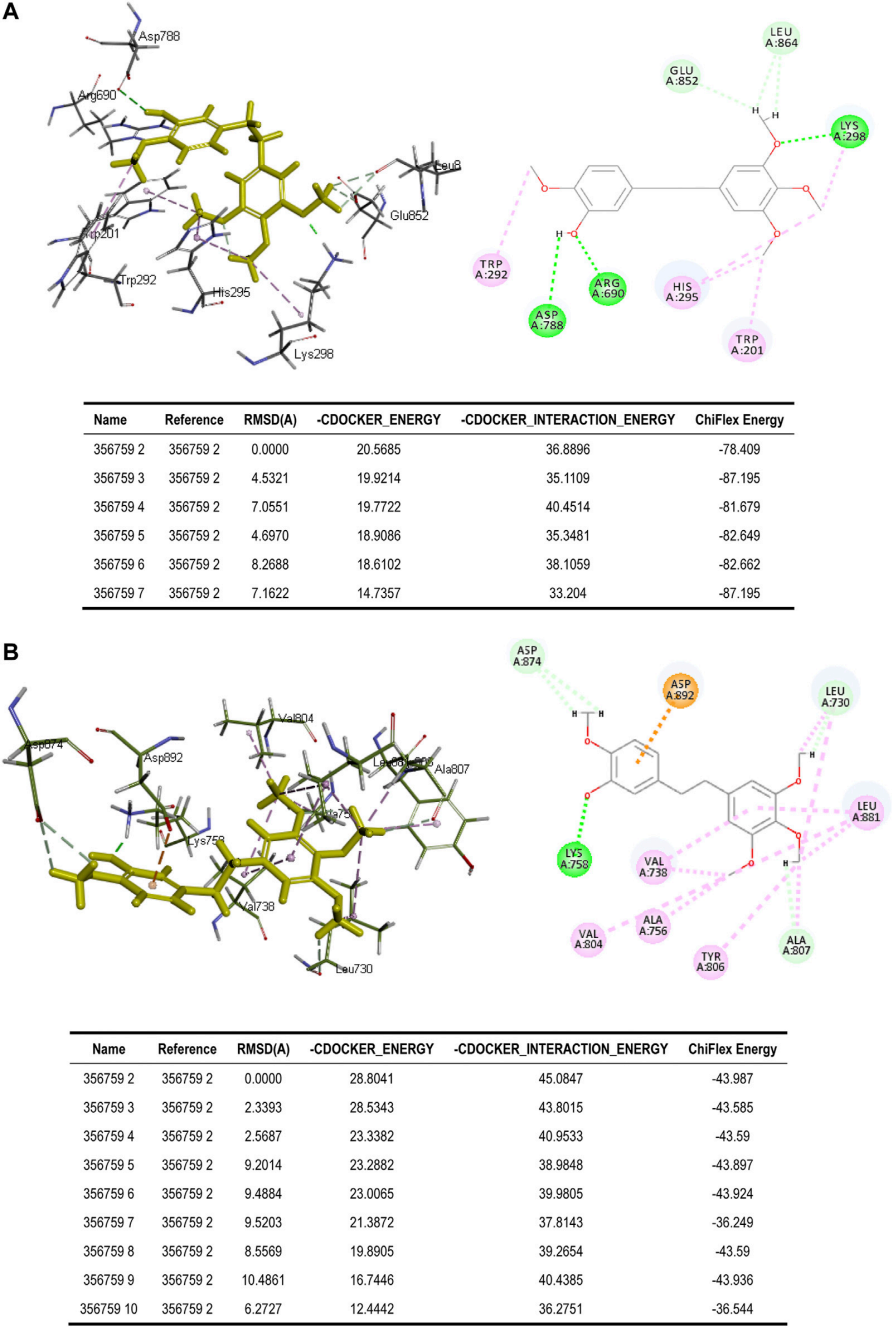
Binding of erianin with **(A)** PI3K and **(B)** RET by molecular docking. Erianin exhibits high binding affinity to PI3K protein *via* sites of Trp292, Asp788, Arg690, His295, Trp201, Lys298, Leu864, and Glu852, and to RET protein *via* sites of Asp874, Asp892, Lys758, Leu730, Leu881, Val738, Val804, Ala756, Tyr806, and Ala807.

**FIGURE 5:**
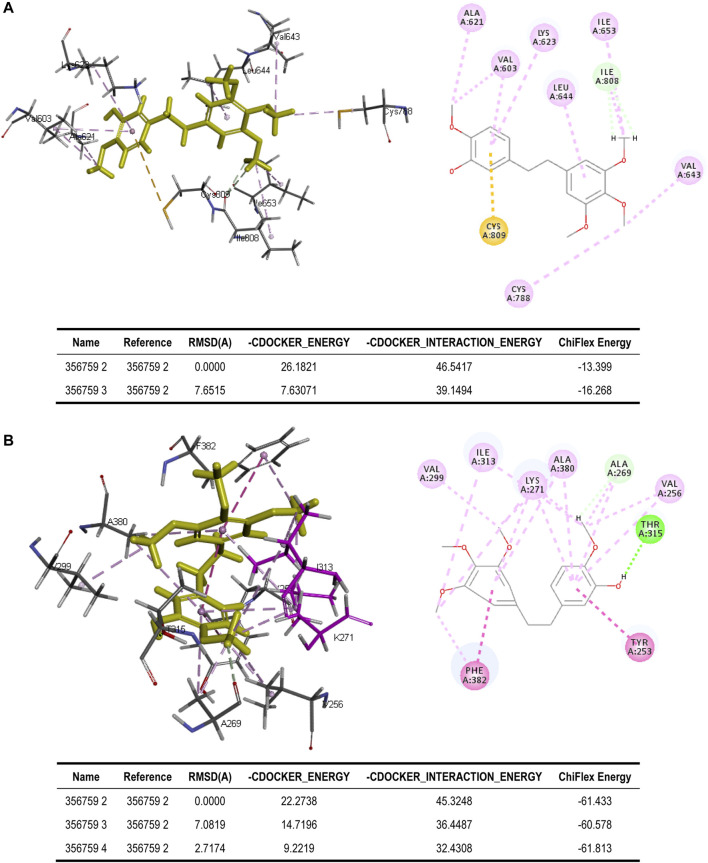
Binding of erianin with **(A)** KIT and **(B)** ABL1 by molecular docking. Erianin exhibits high binding affinity to KIT protein *via* sites of Ala621, Val603, Lys623, Leu644, Ile653, Ile808, Val643, Cys788, and Cys809, and to ABL1 protein *via* sites of Val299, Ile313, Lys271, Ala380, Ala269, Val256, Thr315, Tyr253, and Phe382.

**FIGURE 6:**
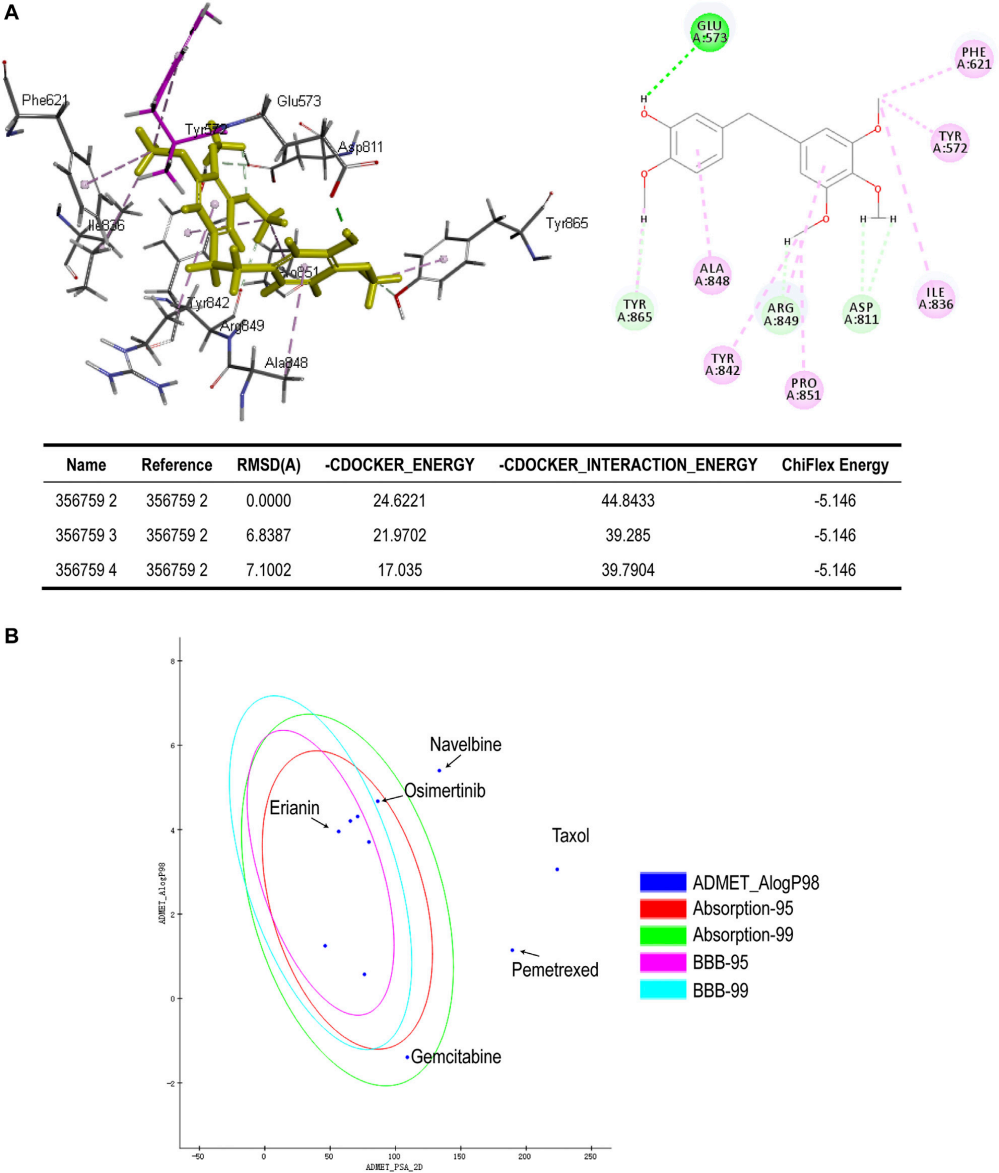
**(A)** Binding of erianin with FLT3 by molecular docking. Erianin exhibits high binding affinity to FLT3 protein *via* sites of Glu573, Phe621, Tyr572, Ile836, Asp811, Pro851, Arg849, Tyr842, Ala848, and Tyr865. **(B)** ADMET plot, plotted by ADMET_PSA_2D vs. ADMET_AlogP98. The dark blue dots represent AlogP98 of each drug. The red and green ellipses represent 95 and 99% confidence intervals of the blood–brain barrier (BBB) permeability model, respectively, and the rose red and light blue ellipses represent 95 and 99% confidence intervals of the human intestinal absorption (HIA) model, respectively.

To better assess the binding ability of erianin to its potential targets, molecular docking was subsequently performed using Discovery Studio. Indeed, as is shown in Figure 4A, erianin exhibits high binding affinity to PI3K protein with the -CDOCKER energy of 20.5685 kcal/mole *via* sites of Trp292, Asp788, Arg690, His295, Trp201, Lys298, Leu864, and Glu852, indicating the high potential of PIK3CA as a target of erianin, which is consistent with the previously reported study (Zhang et al., 2021a). Besides, erianin also shows high binding affinity to RET and KIT in theory with the -CDOCKER energy of 28.8041 kcal/mole and 26.1821 kcal/mole, respectively, as shown in Figure 4B and Figure 5A. Both of the molecular structures of RET and KIT show more than one binding site with erianin, highlighting their potential as targets of erianin. RET and KIT are two proto-oncogenes, whose activation contributes to the development of human cancers, and thus considered attractive targets for specific cancer therapies (Kodama et al., 2005; Abbaspour Babaei et al., 2016; Romei et al., 2016; Ferrara et al., 2018). However, to the best of our knowledge, there is so far no available report confirming the regulation or interaction of erianin with either RET or KIT. Furthermore, ABL1 and FLT3 also show high binding potential with erianin with the -CDOCKER energy of 22.2738 kcal/mole and 24.6221 kcal/mole, respectively, as evaluated by the molecular docking analysis (Figure 5B and Figure 6A). FLT3 is an important cytokine receptor involved in hematopoiesis regulation. It is a common therapeutic target in acute myeloid leukemia (AML). Several inhibitors targeting on FLT3 have been approved for treatment of AML patients with FLT3 mutation such as midostaurin, while others are in preclinical and clinical studies (Wu et al., 2018). ABL1 is a proto-oncogene that encodes a protein tyrosine kinase which plays key roles in a wide variety of cellular processes related to cell growth, survival, and response to oxidative stress. In chronic myeloid leukemia (CML), ABL1 is highly related to BCR–ABL fusion protein and associated with drug sensitivity and patient prognosis (Yang et al., 2020b). Similarly, BCR–ABL1 inhibitors were successively brought into clinical trials for the treatment of patients with CML (Druker et al., 2001; O’Hare et al., 2009). According to the analysis in this study, erianin may serve as a drug candidate for dual inhibition of FLT3 and ABL1 with one stone, indicating its high potential and value in drug development for cancer therapy.

#### 3.2.3 Correlation Analysis Between the Expression of Predicted Targets and Cancer Progression

**FIGURE 7:**
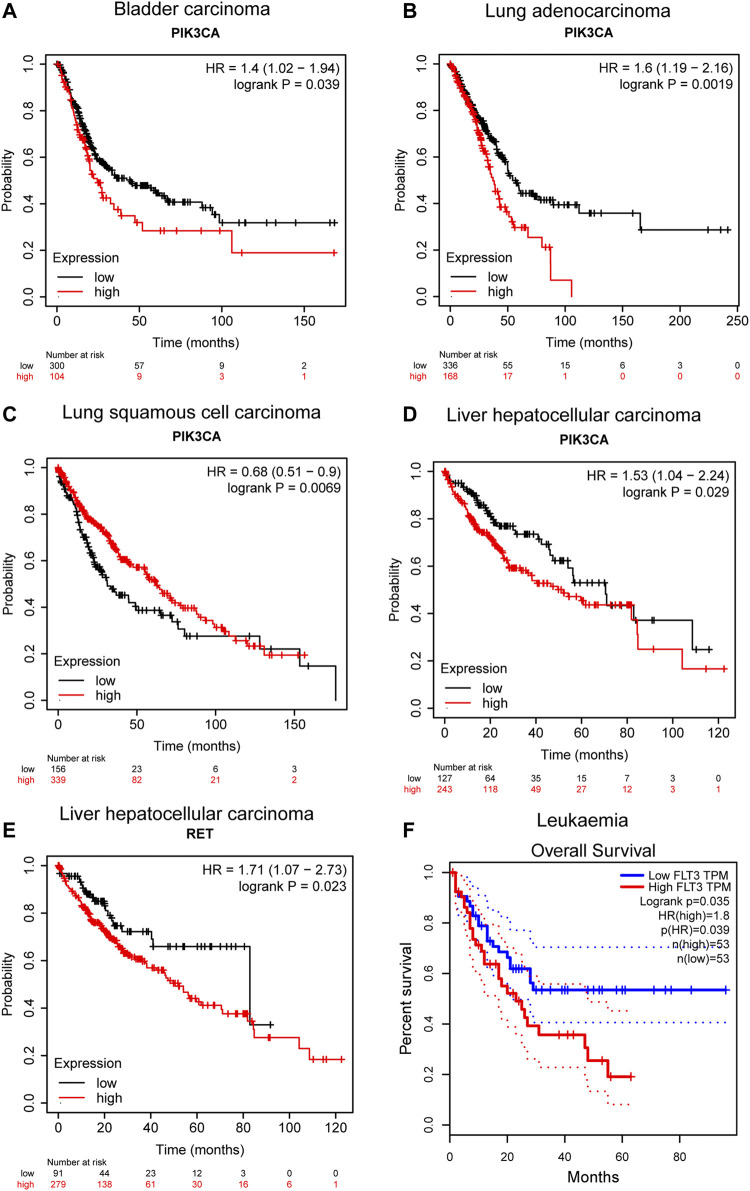
Correlation between predicted target expression and survival prognosis of specific cancers. **(A–E)** KM-plotter and **(F)** GEPIA were used to perform overall survival analyses of different cancers by corresponding gene expression.

Kaplan-Meier plotter (Nagy et al., 2021) and GEPIA (Tang et al., 2017) were used to assess the correlation of the above targets with overall survival in specific cancer. According to the Kaplan-Meier plot, PIK3CA showed potential prognostic significance in bladder carcinoma (*p* = 0.039, *n* = 485), lung adenocarcinoma (*p* = 0.0019, *n* = 601), lung squamous cell carcinoma (*p* = 0.0069, *n* = 632), and liver hepatocellular carcinoma (*p* = 0.029, *n* = 704), and high expression of PIK3CA is associated with poor prognosis in bladder carcinoma, lung adenocarcinoma, and liver hepatocellular carcinoma (Figures 7A–D). PIK3CA encodes the catalytic subunit of PI3K and thus highly correlated with the PI3K/AKT signaling pathway which is considered to play a major role in bladder cancer initiation and progression (Calderaro et al., 2014). A previously reported study has demonstrated that PIK3CA could serve as one of the biomarkers for predicting prognosis and selecting appropriate therapies for patients with bladder cancer in the clinic by genomic DNA analysis of bladder normal and tumor tissues (Kachrilas et al., 2019). It was also found that the increased level of PIK3CA-mutated DNA in urine and plasma is closely associated with later progression and metastasis in bladder cancer by liquid biopsy analysis (Christensen et al., 2017). Strikingly, the TP53/PIK3CA/ATM mutation classifier was recently constructed which can be successfully used for predicting the benefit of immune checkpoint inhibitor therapy for patients with bladder cancer (Pan et al., 2021). Besides, the prognostic value of PIK3CA mutations has also been investigated in lung adenocarcinoma, and the results suggested that PIK3CA mutations are associated with poor prognosis in patients with curative resection, suggesting its significant role in lung adenocarcinoma (Zhang et al., 2013; Song et al., 2016). Interestingly, in lung squamous cell carcinoma, the expression of PIK3CA is positively correlated with prognosis, indicating a cancer-specific prognostic significance of PIK3CA (Figure 7C). Such correlation does not statistically exist in breast cancer, cervical squamous cell carcinoma, and sarcoma as shown in Supplementary Figure S1. In addition, the prognostic potential of RET in liver hepatocellular carcinoma was also observed (*p* = 0.023, *n* = 704), which shows that a high expression of RET in liver hepatocellular carcinoma correlates with a poor prognosis (Figure 7E). This correlation was also proved previously by other researchers, who found that RET mutation is associated with poor prognosis of patients with hepatocellular carcinoma and it potentially serves as a prognostic marker in hepatocellular carcinoma (Ye et al., 2017). Moreover, KIT shows no prognostic significance either in sarcoma or in leukemia when analyzing its correlation with overall survival of sarcoma and leukemia by KM-plotter and GEPIA, respectively (Supplementary Figure S1). Instead, according to the result shown in Figure 7F, the expression of FLT3 may potentially act as a prognostic marker in leukemia (*p* = 0.035, *n* = 106), and the high expression of FLT3 indicates a poor prognosis. This is in line with the previous report, in which overexpression of FLT3 is demonstrated to be a risk factor in leukemia and its mutations confer a poor prognosis (Cheng et al., 2018, Gilliland and Griffin, 2002b; Gilliland and Griffin, 2002a). In fact, FLT3 has already been identified and employed as a viable therapeutic target in AML. The first-generation FLT3 inhibitors, also known as TKIs (tyrosine kinase inhibitors) like sunitinib and sorafenib, are already in clinical use for AML therapy, and the next-generation TKIs with greater specificity for FLT3 are also under clinical investigation (Daver et al., 2019). Taken together, the majority of the predicted targets in this study show high potential and significant value to serve as prognostic factors or functional therapeutic targets in specific cancer. In this regard, erianin may exert anticancer activity in corresponding cancers by targeting or regulating those molecules, highlighting the promising role of erianin in drug development for cancer therapy.

### 3.3 Absorption, Distribution, Metabolism, Excretion, and Toxicity Prediction of Erianin

*In silico* prediction of ADMET (absorption, distribution, metabolism, excretion, and toxicity) is a vital part in the early phases of drug discovery. ADMET parameters of erianin were calculated and computed using ADMET Descriptors and Filter by Lipinski tools available with Discovery Studio. Physical descriptors such as aqueous solubility, blood–brain barrier (BBB) penetration, human intestinal absorption (HIA), hepatotoxicity, cytochrome P450 2D6 inhibition, and plasma protein binding were generated from the analysis. As is shown in Figure 6B, erianin is within the 99% confidence interval, suggesting its reliable prediction. Erianin exhibited satisfactory ADMET properties according to Lipinski’s rule of five (ROF) which estimates the drug-likeness properties of tested compounds. Firstly, the molecular weight of erianin is 318.36, lower than 500 Da and within the acceptable range. H-bond donors and H-bond acceptors, the number of which would affect the BBB permeability and intestinal absorption, are also within the range required (one H-bond donor and five H-bond acceptors in erianin). However, the computed AlogP of erianin is 3.953, suggesting its relatively poor solubility. Besides, the predicted levels of solubility, intestinal absorption, and BBB permeability are 2, 0, and 1, respectively, suggesting its low water solubility, good intestinal absorption, and relatively high BBB penetration (Tables 2, 3). In addition, hepatotoxicity, cytochrome P450 2D6 inhibition, and plasma protein binding parameters of erianin were also included in this paper. Like most chemotherapeutic drugs, erianin was predicted to not be able to inhibit cytochrome P450 2D6 (Table 4). A high plasma protein binding rate was also predicted for erianin, which may facilitate the transport and continuous release of erianin in the blood (Table 5). Besides, erianin may have certain hepatotoxicity, however, lower than osimertinib and pemetrexed as shown in Table 6. Furthermore, the toxicity prediction of erianin was conducted using the “TOPKAT” module in Discovery Studio. Toxicity parameters including rodent carcinogenicity, mutagenicity, aerobic biodegradability, and rat oral LD50 were recorded. According to the prediction reports, erianin exhibits suitable toxicity for drug development with no degradability, mutagenicity, or carcinogenicity (in female mouse), and 6.79 g/kg rat oral LD50.

**TABLE 2:** Blood–brain barrier penetration and human intestinal absorption prediction of erianin and reference drugs.

Compounds	BBB	BBB level	Absorption level	AlogP98	PSA_2D
Erianin	0.173	1 (very high)	0 (good absorption)	3.953	56.535
Carboplatin	−1.184	3 (low)	0 (good absorption)	0.57	76.232
Gemcitabine	—	4 (undefined)	1 (moderate absorption)	−1.394	109.077
Gefitinib	0.109	1 (very high)	0 (good absorption)	4.203	65.474
Erlotinib	0.054	1 (very high)	0 (good absorption)	4.309	71.052
Anlotinib	−0.269	2 (high)	0 (good absorption)	3.705	79.646
Osimertinib	—	4 (undefined)	0 (good absorption)	4.671	86.426
Pemetrexed	—	4 (undefined)	3 (very low absorption)	1.144	189.373
TAX (Taxol)	—	4 (undefined)	3 (very low absorption)	3.055	223.712
Cis-platinum	−0.499	2 (high)	0 (good absorption)	1.246	46.175
Navelbine	—	4 (undefined)	2 (low absorption)	5.397	133.55

**TABLE 3:** Aqueous solubility prediction of erianin and reference drugs.

Compounds	Solubility	Solubility level
Erianin	−4.221	2 (yes, low)
Carboplatin	−0.553	4 (yes, optimal)
Gemcitabine	−0.844	4 (yes, optimal)
Gefitinib	−5.561	2 (yes, low)
Erlotinib	−5.11	2 (yes, low)
Anlotinib	−6.31	1 (no, very low, but possible)
Osimertinib	−5.694	2 (yes, low)
Pemetrexed	−3.488	3 (yes, good)
TAX (Taxol)	−3.515	3 (yes, good)
Cis-platinum	−1.479	4 (yes, optimal)
Navelbine	−6.805	1 (no, very low, but possible)

**TABLE 4:** Cytochrome P450 2D6 inhibitor prediction of erianin and reference drugs.

Compounds	CYP2D6	Prediction	Applicability #MD	Applicability #Mdpvalue
Erianin	−0.338697	FALSE	11.8374	4.17E-03
Carboplatin	−5.29193	FALSE	10.5984	4.53E-02
Gemcitabine	−4.0935	FALSE	15.3695	7.77E-07
Gefitinib	2.41414	TRUE	24.2807	6.84E-17
Erlotinib	−2.88107	FALSE	24.3098	6.37E-17
Anlotinib	−1.94775	FALSE	19.8893	4.74E-12
Osimertinib	−4.75041	FALSE	19.5013	1.32E-11
Pemetrexed	−10.306	FALSE	15.0908	1.61E-06
TAX (Taxol)	−9.84616	FALSE	12.6519	6.87E-04
Cis-platinum	−7.49429	FALSE	17.6396	1.86E-09
Navelbine	−0.936984	FALSE	26.3363	5.09E-19

**TABLE 5:** Plasma protein binding (PPB) rate prediction of erianin and reference drugs.

Compounds	PPB	Prediction	Applicability #MD	Applicability #Mdpvalue
Erianin	−0.938,931	TRUE	8.89311	0.997988
Carboplatin	−7.62992	FALSE	6.8879	1
Gemcitabine	−25.4599	FALSE	10.9394	5.18E-01
Gefitinib	2.80835	TRUE	18.2978	7.72E-17
Erlotinib	0.619653	TRUE	15.3178	1.09E-07
Anlotinib	0.361353	TRUE	13.6617	4.55E-04
Osimertinib	−5.82259	FALSE	19.2739	2.01E-20
Pemetrexed	−15.3292	FALSE	15.4986	3.75E-08
TAX (Taxol)	19.7216	TRUE	15.1283	3.26E-07
Cis-platinum	−5.79062	FALSE	10.9894	4.92E-01
Navelbine	−30.5112	FALSE	17.2142	3.59E-13

**TABLE 6:** Hepatotoxicity prediction of erianin and reference drugs.

Compounds	Hepatotoxic	Prediction	Applicability #MD	Applicability #Mdpvalue
Erianin	0.244421	TRUE	8.92613	4.94E-01
Carboplatin	−3.70565	TRUE	5.69559	0.999998
Gemcitabine	−2.15171	TRUE	8.43163	7.35E-01
Gefitinib	−3.99306	TRUE	14.2139	2.99E-09
Erlotinib	−2.16035	TRUE	13.3441	3.25E-07
Anlotinib	−1.78405	TRUE	13.0744	1.26E-06
Osimertinib	2.59551	TRUE	15.515	1.21E-12
Pemetrexed	1.63974	TRUE	12.5966	1.22E-05
TAX (Taxol)	−9.53069	FALSE	16.426	3.21E-15
Cis-platinum	−6.17439	FALSE	10.0087	8.84E-02
Navelbine	−4.69006	FALSE	16.5462	1.43E-15

## 4 Conclusion

The extensive anticancer activity of erianin in a wide range of cancers has been frequently investigated. The reported mechanisms of action include, but not limit to, induction of apoptosis, cell cycle arrest, and ferroptosis and modulation of immune responses. A thorough understanding of its mechanisms of action is highly necessary and meaningful for the development of erianin as an anticancer agent. According to the prediction and molecular docking results presented in this study, erianin shows high potential to interact with molecules like PIK3CA, RET, KIT, ABL1, and FLT3 in cancers, all of which were found to play important roles in cancer progression. This expands our understanding of action mechanisms of erianin in cancer therapy and provides valuable target candidates and references for future investigation. Besides, drugs designed based on the erianin structure are also getting more and more attention in the research area of cancer therapy. The previously reported erianin derivatives such as ZJU-6 and Ecust004 have shown attractive anticancer activities in cancer models, opening up a new researchable perspective. And combination design of erianin with a nanoparticle drug delivery system to improve the poor solubility of erianin and enhance its anticancer activity could also be a worthy endeavor. However, long way still needs to go before the clinical application of erianin, and efforts are still needed to deeply evaluate the efficacy and safety of erianin *in vitro* and *in vivo*. Particularly, attention should be paid to the possible side effects of erianin such as intestinal hemorrhage and cardiotoxicity.

## Data Availability

The original contributions presented in the study are included in the article/Supplementary Material, further inquiries can be directed to the corresponding authors.
