# Supplementary material for: Anticancer Activity of Erianin: Cancer-Specific Target Prediction Based on Network Pharmacology
Source: Front Mol Biosci. 2022 Mar 17;9:862932. doi: 10.3389/fmolb.2022.862932 (PMC8968680; doi:10.3389/fmolb.2022.862932)
Supplement: Supplementary file 1 [file DataSheet1.docx]

**Anticancer activity of erianin: cancer-specific target prediction based on network pharmacology**

Lili Yan^1,2^, Zhen Zhang^3^, Yanfen Liu^1,2^, Shuyi Ren^1,2^, Zhiyu Zhu^1,2^, Lu Wei^1,2^, Jiao Feng^1,2^, Ting Duan^1,2^, Xueni Sun^1,2*^, Tian Xie^1,2*^, Xinbing Sui^1,2*^

^1^ School of Pharmacy, Hangzhou Normal University, Hangzhou, Zhejiang 311121, China

^2^ Key Laboratory of Elemene Class Anti-Cancer Chinese Medicines; Engineering Laboratory of Development and Application of Traditional Chinese Medicines; Collaborative Innovation Center of Traditional Chinese Medicines of Zhejiang Province, Hangzhou Normal University, Hangzhou, Zhejiang 311121, China

^3^ Department of Orthopedic Surgery, Hangzhou Orthopedic Institute, Affiliated Hangzhou First People's Hospital, Zhejiang University School of Medicine, Hangzhou, Zhejiang 310003, China

^*^Corresponding authors to: [hzzju@hznu.edu.cn](mailto:hzzju@hznu.edu.cn) (X.S.), [xbs@hznu.edu.cn](mailto:xbs@hznu.edu.cn) (T.X.), [xnsun@hznu.edu.cn](mailto:xnsun@hznu.edu.cn) (X.S.)


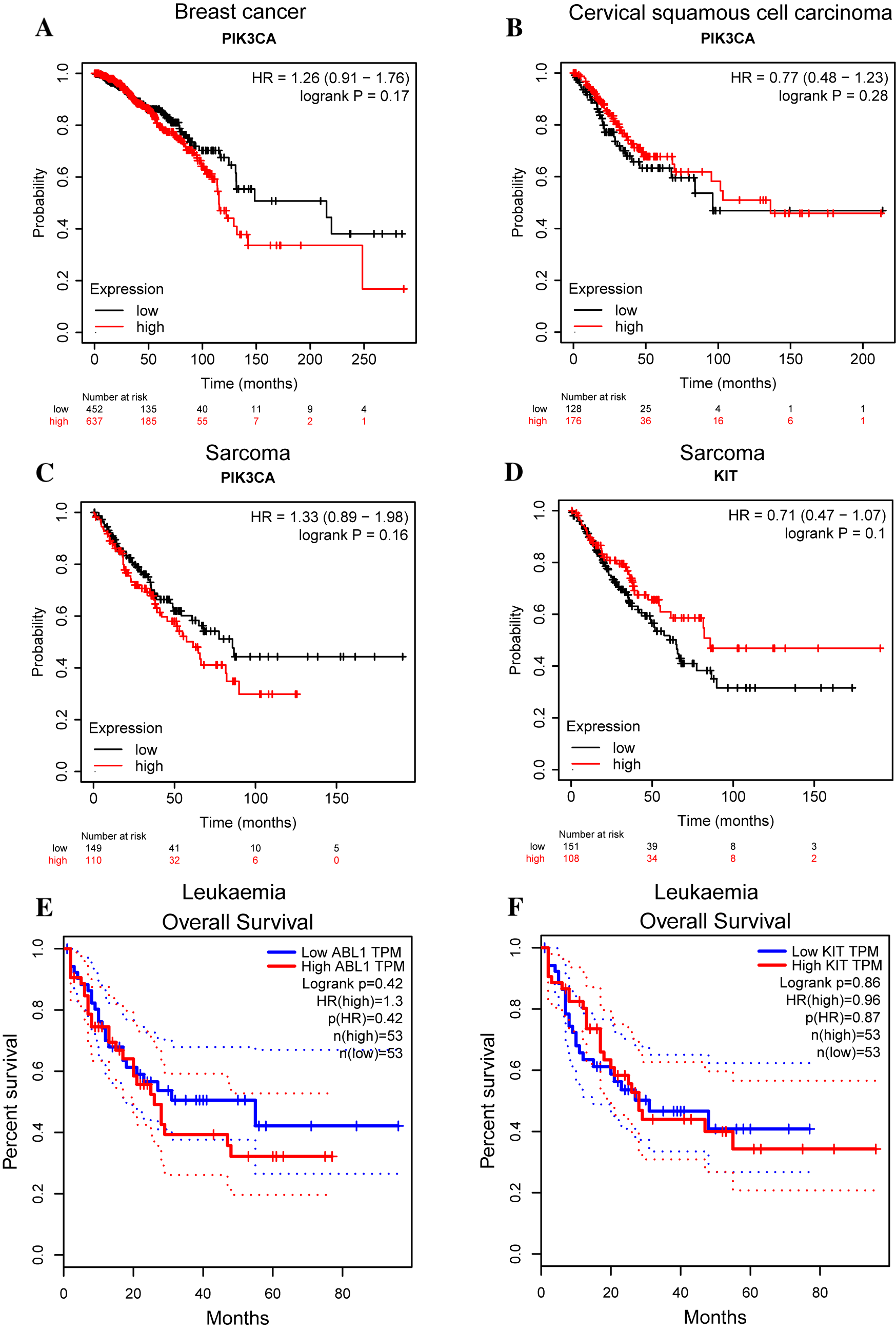


**Figure S1.** Correlation analysis of the expression of PIK3CA, KIT, and ABL1 with overall survival of specific cancer. Figure S1A-D were obtained from KM-plotter and Figure S1E-F were obtained from GEPIA.

**Table S1.** Predicted targets of erianin obtained from Swiss Target Prediction database.

| **Predicted targets** | **Common name** | **Uniprot ID** | **ChEMBL ID** | **Targets Class** | **Probability*** |
| --- | --- | --- | --- | --- | --- |
| Calmodulin | CALM1 | P62158 | CHEMBL6093 | Unclassified protein | 0.243267 |
| Cytochrome P450 19A1 | CYP19A1 | P11511 | CHEMBL1978 | Cytochrome P450 | 0.18493 |
| Tubulin beta-1 chain | TUBB1 | Q9H4B7 | CHEMBL1915 | Structural protein | 0.109946 |
| Arachidonate 15 -lipoxygenase | ALOX15 | P16050 | CHEMBL2903 | Enzyme | 0.101614 |
| HMG-CoA reductase | HMGCR | P04035 | CHEMBL402 | Oxidoreductase | 0.101614 |
| Glucocorticoid receptor | NR3C1 | P04150 | CHEMBL2034 | Nuclear receptor | 0.101614 |
| Serotonin 2a (5-HT2a) receptor | HTR2A | P28223 | CHEMBL224 | Family A G protein-coupled receptor | 0.101614 |
| Insulin-like growth factor I receptor | IGF1R | P08069 | CHEMBL1957 | Kinase | 0.101614 |
| Arachidonate 12 -lipoxygenase | ALOX12 | P18054 | CHEMBL3687 | Enzyme | 0.101614 |
| P-glycoprotein 1 | ABCB1 | P08183 | CHEMBL4302 | Primary active transporter | 0.101614 |
| Transthyretin | TTR | P02766 | CHEMBL3194 | Secreted protein | 0.101614 |
| Kinesin-1 heavy chain/ Tyrosine-protein kinase receptor RET | RET | P07949 | CHEMBL2041 | Kinase | 0.101614 |
| Receptor protein-tyrosine kinase erbB-2 | ERBB2 | P04626 | CHEMBL1824 | Kinase | 0.101614 |
| Serine/threonine-protein kinase PIM1 | PIM1 | P11309 | CHEMBL2147 | Kinase | 0.101614 |
| Interleukin-8 receptor B | CXCR2 | P25025 | CHEMBL2434 | Family A G protein-coupled receptor | 0.101614 |
| Cytochrome P450 11B2 | CYP11B2 | P19099 | CHEMBL2722 | Cytochrome P450 | 0.101614 |
| Nitric oxide synthase, inducible | NOS2 | P35228 | CHEMBL4481 | Enzyme | 0.101614 |
| Indoleamine 2,3-dioxygenase | IDO1 | P14902 | CHEMBL4685 | Enzyme | 0.101614 |
| Huntingtin | HTT | P42858 | CHEMBL5514 | Unclassified protein | 0.101614 |
| Equilibrative nucleoside transporter 1 | SLC29A1 | Q99808 | CHEMBL1997 | Electrochemical transporter | 0.101614 |
| Caspase-6 | CASP6 | P55212 | CHEMBL3308 | Protease | 0.101614 |
| Egl nine homolog 1 | EGLN1 | Q9GZT9 | CHEMBL5697 | Oxidoreductase | 0.101614 |
| Vascular endothelial growth factor receptor 1 | FLT1 | P17948 | CHEMBL1868 | Kinase | 0.101614 |
| PI3-kinase p85-alpha subunit | PIK3R1 | P27986 | CHEMBL2506 | Enzyme | 0.101614 |
| Cyclin-dependent kinase 2 | CDK2 | P24941 | CHEMBL301 | Kinase | 0.101614 |
| Ephrin type-B receptor 2 | EPHB2 | P29323 | CHEMBL3290 | Kinase | 0.101614 |
| c-Jun N-terminal kinase 3 | MAPK10 | P53779 | CHEMBL2637 | Kinase | 0.101614 |
| Rho-associated protein kinase 2 | ROCK2 | O75116 | CHEMBL2973 | Kinase | 0.101614 |
| Rho-associated protein kinase 1 | ROCK1 | Q13464 | CHEMBL3231 | Kinase | 0.101614 |
| Glutaminyl-peptide cyclotransferase | QPCT | Q16769 | CHEMBL4508 | Enzyme | 0.101614 |
| Phosphodiesterase 10A | PDE10A | Q9Y233 | CHEMBL4409 | Phosphodiesterase | 0.101614 |
| Adenosine A1 receptor | ADORA1 | P30542 | CHEMBL226 | Family A G protein-coupled receptor | 0.101614 |
| Adenosine A2a receptor | ADORA2A | P29274 | CHEMBL251 | Family A G protein-coupled receptor | 0.101614 |
| Adenosine A2b receptor | ADORA2B | P29275 | CHEMBL255 | Family A G protein-coupled receptor | 0.101614 |
| Platelet-derived growth factor receptor beta | PDGFRB | P09619 | CHEMBL1913 | Kinase | 0.101614 |
| Tyrosine-protein kinase JAK3 | JAK3 | P52333 | CHEMBL2148 | Kinase | 0.101614 |
| Tyrosine-protein kinase JAK2 | JAK2 | O60674 | CHEMBL2971 | Kinase | 0.101614 |
| Tyrosine-protein kinase ABL | ABL1 | P00519 | CHEMBL1862 | Kinase | 0.101614 |
| Serine/threonine-protein kinase mTOR | MTOR | P42345 | CHEMBL2842 | Kinase | 0.101614 |
| PI3-kinase p110-delta subunit | PIK3CD | O00329 | CHEMBL3130 | Enzyme | 0.101614 |
| DNA-dependent protein kinase | PRKDC | P78527 | CHEMBL3142 | Kinase | 0.101614 |
| PI3-kinase p110-beta subunit | PIK3CB | P42338 | CHEMBL3145 | Enzyme | 0.101614 |
| Tyrosine-protein kinase HCK | HCK | P08631 | CHEMBL3234 | Kinase | 0.101614 |
| PI3-kinase p110-gamma subunit | PIK3CG | P48736 | CHEMBL3267 | Enzyme | 0.101614 |
| Heat shock protein HSP 90-alpha | HSP90AA1 | P07900 | CHEMBL3880 | Other cytosolic protein | 0.101614 |
| PI3-kinase p110-alpha subunit | PIK3CA | P42336 | CHEMBL4005 | Enzyme | 0.101614 |
| Ephrin receptor | EPHB4 | P54760 | CHEMBL5147 | Kinase | 0.101614 |
| Myosin light chain kinase, smooth muscle | MYLK | Q15746 | CHEMBL2428 | Kinase | 0.101614 |
| Serine/threonine-protein kinase B-raf | BRAF | P15056 | CHEMBL5145 | Kinase | 0.101614 |
| Quinone reductase 2 | NQO2 | P16083 | CHEMBL3959 | Enzyme | 0.101614 |
| Serine/threonine-protein kinase RAF | RAF1 | P04049 | CHEMBL1906 | Kinase | 0.101614 |
| Cyclin-dependent kinase 4 | CDK4 | P11802 | CHEMBL331 | Kinase | 0.101614 |
| Lysine-specific histone demethylase 1 | KDM1A | O60341 | CHEMBL6136 | Eraser | 0.101614 |
| Nitric-oxide synthase, brain | NOS1 | P29475 | CHEMBL3568 | Enzyme | 0.101614 |
| Fibroblast growth factor receptor 1 | FGFR1 | P11362 | CHEMBL3650 | Kinase | 0.101614 |
| MAP kinase-interacting serine/threonine-protein kinase MNK1 | MKNK1 | Q9BUB5 | CHEMBL4718 | Kinase | 0.101614 |
| Serotonin transporter | SLC6A4 | P31645 | CHEMBL228 | Electrochemical transporter | 0.101614 |
| PI4-kinase beta subunit | PI4KB | Q9UBF8 | CHEMBL3268 | Enzyme | 0.101614 |
| Estradiol 17-beta-dehydrogenase 3 | HSD17B3 | P37058 | CHEMBL4234 | Enzyme | 0.101614 |
| Serotonin 1a (5-HT1a) receptor | HTR1A | P08908 | CHEMBL214 | Family A G protein-coupled receptor | 0.101614 |
| Gamma-amino-N-butyrate transaminase (by homology) | ABAT | P80404 | CHEMBL2044 | Transferase | 0.101614 |
| Beta secretase 2 | BACE2 | Q9Y5Z0 | CHEMBL2525 | Protease | 0.101614 |
| Serine/threonine-protein kinase Chk2 | CHEK2 | O96017 | CHEMBL2527 | Kinase | 0.101614 |
| Histone chaperone ASF1A | ASF1A | Q9Y294 | CHEMBL3392950 | Unclassified protein | 0.101614 |
| Beta-secretase 1 | BACE1 | P56817 | CHEMBL4822 | Protease | 0.101614 |
| Cyclin-dependent kinase 2/cyclin E | CCNE2 CDK2 CCNE1 | O96020 P24941 P24864 | CHEMBL2094126 | Other cytosolic protein | 0.101614 |
| Cystinyl aminopeptidase | LNPEP | Q9UIQ6 | CHEMBL2693 | Protease | 0.101614 |
| Glycogen synthase kinase-3 alpha | GSK3A | P49840 | CHEMBL2850 | Kinase | 0.101614 |
| Poly [ADP-ribose] polymerase-1 | PARP1 | P09874 | CHEMBL3105 | Enzyme | 0.101614 |
| Serine/threonine-protein kinase Chk1 | CHEK1 | O14757 | CHEMBL4630 | Kinase | 0.101614 |
| Arachidonate 5-lipoxygenase | ALOX5 | P09917 | CHEMBL215 | Oxidoreductase | 0.101614 |
| Cyclin-dependent kinase 4/cyclin D1 | CCND1 CDK4 | P24385 P11802 | CHEMBL1907601 | Kinase | 0.101614 |
| Cyclin-dependent kinase 1/cyclin B1 | CDK1 CCNB1 | P06493 P14635 | CHEMBL1907602 | Other cytosolic protein | 0.101614 |
| Cyclin-dependent kinase 2/cyclin E1 | CCNE1 CDK2 | P24864 P24941 | CHEMBL1907605 | Kinase | 0.101614 |
| Cytochrome P450 11B1 | CYP11B1 | P15538 | CHEMBL1908 | Cytochrome P450 | 0.101614 |
| Cyclin-dependent kinase 4/cyclin D | CCND3 CCND1 CDK4 CCND2 | P30281 P24385 P11802 P30279 | CHEMBL2095942 | Other cytosolic protein | 0.101614 |
| MAP kinase-activated protein kinase 2 | MAPKAPK2 | P49137 | CHEMBL2208 | Kinase | 0.101614 |
| Acetylcholinesterase | ACHE | P22303 | CHEMBL220 | Hydrolase | 0.101614 |
| Cyclin-dependent kinase 1/cyclin B | CCNB3 CDK1 CCNB1 CCNB2 | Q8WWL7 P06493 P14635 O95067 | CHEMBL2094127 | Other cytosolic protein | 0.101614 |
| Phosphodiesterase 2A | PDE2A | O00408 | CHEMBL2652 | Phosphodiesterase | 0.101614 |
| Fatty acid desaturase 1 | FADS1 | O60427 | CHEMBL5840 | Enzyme | 0.101614 |
| Stem cell growth factor receptor | KIT | P10721 | CHEMBL1936 | Kinase | 0.101614 |
| Vascular endothelial growth factor receptor 3 | FLT4 | P35916 | CHEMBL1955 | Kinase | 0.101614 |
| Tyrosine-protein kinase receptor FLT3 | FLT3 | P36888 | CHEMBL1974 | Kinase | 0.101614 |
| Platelet-derived growth factor receptor alpha | PDGFRA | P16234 | CHEMBL2007 | Kinase | 0.101614 |
| Cyclin-dependent kinase 2/cyclin A | CDK2 CCNA1 CCNA2 | P24941 P78396 P20248 | CHEMBL2094128 | Other cytosolic protein | 0.101614 |
| Serine/threonine-protein kinase PLK4 | PLK4 | O00444 | CHEMBL3788 | Kinase | 0.101614 |
| Tyrosine-protein kinase TIE-2 | TEK | Q02763 | CHEMBL4128 | Kinase | 0.101614 |
| Serine/threonine-protein kinase Aurora-A | AURKA | O14965 | CHEMBL4722 | Kinase | 0.101614 |
| Estrogen receptor alpha | ESR1 | P03372 | CHEMBL206 | Nuclear receptor | 0.101614 |
| Estrogen receptor beta | ESR2 | Q92731 | CHEMBL242 | Nuclear receptor | 0.101614 |
| Thromboxane-A synthase | TBXAS1 | P24557 | CHEMBL1835 | Cytochrome P450 | 0.101614 |
| Melatonin receptor 1A | MTNR1A | P48039 | CHEMBL1945 | Family A G protein-coupled receptor | 0.101614 |
| Melatonin receptor 1B | MTNR1B | P49286 | CHEMBL1946 | Family A G protein-coupled receptor | 0.101614 |
| Cyclin-dependent kinase 5/CDK5 activator 1 | CDK5R1 CDK5 | Q15078 Q00535 | CHEMBL1907600 | Kinase | 0.101614 |
| Dual-specificity tyrosine-phosphorylation regulated kinase 1A | DYRK1A | Q13627 | CHEMBL2292 | Kinase | 0.101614 |
| Cyclooxygenase-2 | PTGS2 | P35354 | CHEMBL230 | Oxidoreductase | 0.101614 |
| Corticotropin releasing factor receptor 1 | CRHR1 | P34998 | CHEMBL1800 | Family B G protein-coupled receptor | 0.101614 |
| Serine/threonine-protein kinase PIM2 | PIM2 | Q9P1W9 | CHEMBL4523 | Kinase | 0.101614 |
| Serine/threonine-protein kinase PIM3 | PIM3 | Q86V86 | CHEMBL5407 | Kinase | 0.101614 |

**Table S2.** A list of potential therapeutic targets predicted in lung cancer, cervical cancer, bladder cancer, colorectal cancer and breast cancer. Targets are given as entrez gene ID.

| **No.** | **lung cancer** | **cervical cancer** | **bladder cancer** | **colorectal cancer** | **breast cancer** |
| --- | --- | --- | --- | --- | --- |
| 1 | 100187710 | 100126794 | 100126794 | 100126794 | 100126794 |
| 2 | 100187711 | 100187710 | 100187710 | 100187710 | 100187710 |
| 3 | 100188769 | 100187711 | 100187711 | 100187711 | 100187711 |
| 4 | 100188789 | 100188769 | 100188769 | 100188769 | 100188769 |
| 5 | 100188809 | 100188789 | 100188789 | 100188789 | 100188789 |
| 6 | 100188834 | 100188809 | 100188809 | 100188809 | 100188809 |
| 7 | 100188841 | 100188834 | 100188834 | 100188834 | 100188834 |
| 8 | 100188867 | 100188841 | 100188841 | 100188841 | 100188841 |
| 9 | 100188868 | 100188867 | 100188867 | 100188867 | 100188867 |
| 10 | 100188887 | 100188868 | 100188868 | 100188868 | 100188868 |
| 11 | 100271687 | 100188887 | 100188887 | 100188887 | 100188887 |
| 12 | 100271690 | 100270680 | 100270680 | 100270680 | 100270680 |
| 13 | 100271692 | 100271687 | 100271687 | 100271687 | 100271687 |
| 14 | 100271693 | 100271690 | 100271690 | 100271690 | 100271690 |
| 15 | 100302516 | 100271692 | 100271692 | 100271692 | 100271692 |
| 16 | 100329169 | 100271693 | 100271693 | 100271693 | 100271693 |
| 17 | 10056 | 100287082 | 100287082 | 100287082 | 100287082 |
| 18 | 100682395 | 100329169 | 100329169 | 100329169 | 100329169 |
| 19 | 1029 | 100505495 | 100505495 | 100505495 | 100505495 |
| 20 | 10395 | 100505994 | 100505994 | 100505994 | 100505994 |
| 21 | 10481 | 100507056 | 100507056 | 100507056 | 100507056 |
| 22 | 10568 | 100682395 | 100682395 | 100526820 | 100682395 |
| 23 | 11200 | 100750225 | 100750225 | 100682395 | 100750225 |
| 24 | 1136 | 100874054 | 100874054 | 100750225 | 100874054 |
| 25 | 1138 | 101669762 | 101669762 | 100874054 | 101669762 |
| 26 | 125 | 101669767 | 101669767 | 101669762 | 101669767 |
| 27 | 1314 | 101805488 | 101805488 | 101669767 | 101805488 |
| 28 | 1316 | 101867536 | 101867536 | 101805488 | 101867536 |
| 29 | 1326 | 1029 | 1029 | 101867536 | 10286 |
| 30 | 1373 | 103021164 | 103021164 | 1029 | 1029 |
| 31 | 1438 | 103021165 | 103021165 | 103021164 | 103021164 |
| 32 | 1439 | 103164619 | 103164619 | 103021165 | 103021165 |
| 33 | 1499 | 10395 | 10395 | 103164619 | 103164619 |
| 34 | 1548 | 104472713 | 104472713 | 10395 | 10395 |
| 35 | 1630 | 104797537 | 104797537 | 104472713 | 104472713 |
| 36 | 1728 | 10481 | 10481 | 104797537 | 104797537 |
| 37 | 1956 | 10904 | 10904 | 10481 | 10481 |
| 38 | 1981 | 11200 | 11200 | 10904 | 10904 |
| 39 | 201163 | 1136 | 1136 | 11200 | 11200 |
| 40 | 2033 | 1138 | 116093 | 1136 | 1136 |
| 41 | 2048 | 116093 | 118425 | 1138 | 1138 |
| 42 | 2064 | 118425 | 120376 | 116093 | 116093 |
| 43 | 207 | 120376 | 125 | 118425 | 118425 |
| 44 | 2074 | 125 | 1316 | 120376 | 120376 |
| 45 | 2099 | 1316 | 1326 | 125 | 125 |
| 46 | 21 | 1326 | 1499 | 1316 | 1316 |
| 47 | 217 | 1499 | 1548 | 1326 | 1326 |
| 48 | 2193 | 1548 | 1601 | 1499 | 1499 |
| 49 | 2261 | 1601 | 1630 | 1548 | 1548 |
| 50 | 2263 | 1630 | 1728 | 1601 | 1601 |
| 51 | 2264 | 1728 | 1956 | 1630 | 1630 |
| 52 | 2271 | 1956 | 1981 | 1728 | 1728 |
| 53 | 2294 | 1981 | 201163 | 1956 | 1956 |
| 54 | 23022 | 201163 | 2017 | 1981 | 1981 |
| 55 | 2304 | 2017 | 2033 | 201163 | 201163 |
| 56 | 23301 | 2033 | 203413 | 2017 | 2017 |
| 57 | 23328 | 203413 | 2048 | 2033 | 2033 |
| 58 | 25788 | 2048 | 2064 | 203413 | 203413 |
| 59 | 260431 | 2064 | 207 | 2048 | 2048 |
| 60 | 27030 | 207 | 2074 | 2064 | 2064 |
| 61 | 285282 | 2074 | 2099 | 207 | 207 |
| 62 | 2956 | 2099 | 217 | 2074 | 2074 |
| 63 | 3026 | 217 | 220064 | 2099 | 2099 |
| 64 | 3161 | 220064 | 2261 | 217 | 217 |
| 65 | 3162 | 2261 | 2263 | 220064 | 220064 |
| 66 | 324 | 2263 | 2264 | 2261 | 2261 |
| 67 | 3265 | 2264 | 2271 | 2263 | 2263 |
| 68 | 347747 | 2271 | 23022 | 2264 | 2264 |
| 69 | 3490 | 23022 | 2304 | 2271 | 2271 |
| 70 | 3553 | 2304 | 23119 | 23022 | 23022 |
| 71 | 3557 | 23119 | 23301 | 2304 | 2304 |
| 72 | 356 | 23301 | 23328 | 23119 | 23119 |
| 73 | 3659 | 23328 | 255082 | 23301 | 23301 |
| 74 | 367 | 255082 | 255313 | 23328 | 23328 |
| 75 | 3675 | 255313 | 256309 | 255082 | 255082 |
| 76 | 374393 | 256309 | 25788 | 255313 | 255313 |
| 77 | 3777 | 25788 | 27030 | 256309 | 256309 |
| 78 | 3845 | 27030 | 285282 | 25788 | 25788 |
| 79 | 4072 | 285282 | 285782 | 27030 | 27030 |
| 80 | 408259 | 285782 | 286205 | 285282 | 285282 |
| 81 | 408260 | 286205 | 2956 | 285782 | 285782 |
| 82 | 4089 | 2956 | 3026 | 286205 | 286205 |
| 83 | 4092 | 3026 | 3090 | 2956 | 2956 |
| 84 | 4093 | 3090 | 3161 | 3026 | 3026 |
| 85 | 4141 | 3161 | 324 | 3090 | 3090 |
| 86 | 4163 | 324 | 3265 | 3161 | 3161 |
| 87 | 4221 | 3265 | 347747 | 324 | 324 |
| 88 | 4292 | 347747 | 3553 | 3265 | 3265 |
| 89 | 4312 | 3553 | 3557 | 347747 | 347747 |
| 90 | 4353 | 3557 | 356 | 3553 | 3553 |
| 91 | 440275 | 356 | 360219 | 3557 | 3557 |
| 92 | 4436 | 360219 | 3659 | 356 | 356 |
| 93 | 4477 | 3659 | 367 | 360219 | 360219 |
| 94 | 450093 | 367 | 3845 | 3659 | 3659 |
| 95 | 450095 | 3845 | 399948 | 367 | 367 |
| 96 | 4595 | 399948 | 400500 | 3845 | 3845 |
| 97 | 4601 | 400500 | 401237 | 399948 | 399948 |
| 98 | 463 | 401237 | 4072 | 400500 | 400500 |
| 99 | 472 | 4072 | 408259 | 401237 | 401237 |
| 100 | 4835 | 408259 | 408260 | 4072 | 4072 |
| 101 | 4893 | 408260 | 4089 | 408259 | 408259 |
| 102 | 4978 | 4089 | 4092 | 408260 | 408260 |
| 103 | 5002 | 4092 | 4163 | 4089 | 4089 |
| 104 | 5071 | 4163 | 4292 | 4092 | 4092 |
| 105 | 5073 | 4292 | 4353 | 4163 | 4163 |
| 106 | 50970 | 4353 | 4436 | 4292 | 4292 |
| 107 | 5157 | 4436 | 4477 | 4353 | 4353 |
| 108 | 51750 | 4477 | 450093 | 4436 | 4436 |
| 109 | 5245 | 450093 | 4595 | 4477 | 4477 |
| 110 | 5290 | 4595 | 4601 | 450093 | 450093 |
| 111 | 5320 | 4601 | 463 | 4595 | 4595 |
| 112 | 5395 | 463 | 472 | 4601 | 4601 |
| 113 | 5424 | 472 | 4835 | 463 | 463 |
| 114 | 5426 | 4835 | 4893 | 472 | 472 |
| 115 | 545 | 4893 | 4978 | 4835 | 4835 |
| 116 | 54894 | 4978 | 5002 | 4893 | 4893 |
| 117 | 54938 | 5002 | 50514 | 4978 | 4978 |
| 118 | 5519 | 50514 | 50652 | 5002 | 5002 |
| 119 | 55596 | 50652 | 5071 | 50514 | 50514 |
| 120 | 56160 | 5071 | 50970 | 50652 | 50652 |
| 121 | 5728 | 50970 | 5157 | 5071 | 5071 |
| 122 | 574048 | 5157 | 5245 | 50970 | 50970 |
| 123 | 57522 | 5245 | 5290 | 5157 | 5157 |
| 124 | 5782 | 5290 | 5320 | 5245 | 5245 |
| 125 | 5795 | 5320 | 5395 | 5290 | 5290 |
| 126 | 580 | 5395 | 5424 | 5320 | 5320 |
| 127 | 581 | 5424 | 5426 | 5395 | 5395 |
| 128 | 5888 | 5426 | 545 | 5424 | 5424 |
| 129 | 5889 | 545 | 54894 | 5426 | 5426 |
| 130 | 5892 | 54894 | 5519 | 545 | 545 |
| 131 | 5925 | 5519 | 55259 | 54894 | 54894 |
| 132 | 595 | 55259 | 5728 | 5519 | 5519 |
| 133 | 6041 | 5728 | 574048 | 55259 | 55259 |
| 134 | 60528 | 574048 | 57522 | 5728 | 55653 |
| 135 | 619402 | 57522 | 5782 | 574048 | 5618 |
| 136 | 6439 | 5782 | 5795 | 57522 | 5728 |
| 137 | 6440 | 5795 | 580 | 5782 | 574048 |
| 138 | 659 | 580 | 581 | 5795 | 57522 |
| 139 | 6714 | 581 | 5888 | 580 | 5782 |
| 140 | 672 | 5888 | 5889 | 581 | 5792 |
| 141 | 673 | 5889 | 5892 | 5888 | 5795 |
| 142 | 675 | 5892 | 5925 | 5889 | 580 |
| 143 | 6790 | 5925 | 59351 | 5892 | 581 |
| 144 | 6794 | 59351 | 595 | 5925 | 5888 |
| 145 | 6893 | 595 | 6041 | 59351 | 5889 |
| 146 | 699 | 6041 | 60528 | 595 | 5892 |
| 147 | 701 | 60528 | 619402 | 6041 | 5925 |
| 148 | 7012 | 619402 | 641654 | 60528 | 59351 |
| 149 | 7015 | 641654 | 643311 | 619402 | 595 |
| 150 | 7040 | 643311 | 652995 | 641654 | 6041 |
| 151 | 7048 | 652995 | 653282 | 643311 | 60528 |
| 152 | 7080 | 653282 | 6623 | 643911 | 619402 |
| 153 | 7097 | 6623 | 6714 | 652995 | 641654 |
| 154 | 7157 | 6714 | 672 | 653282 | 643311 |
| 155 | 727897 | 672 | 673 | 6623 | 652995 |
| 156 | 729238 | 673 | 675 | 6714 | 6623 |
| 157 | 7517 | 675 | 6790 | 672 | 6714 |
| 158 | 7834 | 6762 | 6794 | 673 | 672 |
| 159 | 7864 | 6790 | 699 | 675 | 673 |
| 160 | 79651 | 6794 | 701 | 6790 | 675 |
| 161 | 79695 | 699 | 7031 | 6794 | 6790 |
| 162 | 79728 | 701 | 7048 | 699 | 6794 |
| 163 | 8313 | 7031 | 7080 | 701 | 699 |
| 164 | 8379 | 7048 | 7097 | 7031 | 701 |
| 165 | 83990 | 7080 | 7157 | 7048 | 7031 |
| 166 | 841 | 7097 | 727677 | 7080 | 7048 |
| 167 | 843 | 7157 | 728036 | 7097 | 7080 |
| 168 | 8438 | 727677 | 728042 | 7157 | 7097 |
| 169 | 8493 | 728036 | 728049 | 727677 | 7157 |
| 170 | 857 | 728042 | 728062 | 728036 | 727677 |
| 171 | 91 | 728049 | 728072 | 728042 | 728606 |
| 172 | 9496 | 728062 | 728606 | 728049 | 7517 |
| 173 | 9566 | 728606 | 7517 | 728606 | 7834 |
| 174 | 9821 | 7517 | 7834 | 7517 | 7864 |
| 175 | 999 | 7834 | 7864 | 7834 | 79651 |
| 176 |  | 7864 | 79651 | 7864 | 79695 |
| 177 |  | 79651 | 79695 | 79651 | 79728 |
| 178 |  | 79695 | 79728 | 79695 | 7982 |
| 179 |  | 79728 | 8099 | 79728 | 8099 |
| 180 |  | 8099 | 81552 | 8099 | 81552 |
| 181 |  | 81552 | 8202 | 81552 | 8202 |
| 182 |  | 8202 | 8313 | 8202 | 8313 |
| 183 |  | 8313 | 8379 | 8313 | 8379 |
| 184 |  | 8379 | 83990 | 8379 | 83990 |
| 185 |  | 83990 | 841 | 83990 | 841 |
| 186 |  | 841 | 8412 | 841 | 8412 |
| 187 |  | 8412 | 843 | 8412 | 843 |
| 188 |  | 843 | 8438 | 843 | 8438 |
| 189 |  | 8438 | 8493 | 8438 | 8493 |
| 190 |  | 8493 | 8826 | 8493 | 8537 |
| 191 |  | 8826 | 91 | 8826 | 8826 |
| 192 |  | 91 | 9821 | 91 | 91 |
| 193 |  | 9821 | 9940 | 9821 | 9821 |
| 194 |  | 9940 | 999 | 9940 | 9940 |
| 195 |  | 999 |  | 999 | 999 |

**Table S3.** A list of potential therapeutic targets predicted in oral squamous cell carcinoma, osteosarcoma, leukemia, nasopharyngeal carcinoma and hepatocellular. Targets are given as entrez gene ID.

| **No.** | **oral squamous cell carcinoma** | **Osteosarcoma** | **Leukemia** | **Nasopharyngeal carcinoma** | **Hepatocellular carcinoma** |
| --- | --- | --- | --- | --- | --- |
| 1 | 100128553 | 100128553 | 100188791 | 100302522 | 100302522 |
| 2 | 10015 | 10015 | 100270642 | 100307118 | 100307118 |
| 3 | 10016 | 10016 | 100270643 | 100307119 | 100307119 |
| 4 | 100307118 | 100307118 | 100270644 | 100307120 | 100307120 |
| 5 | 100307119 | 100307119 | 100310785 | 100307121 | 100307121 |
| 6 | 100307120 | 100307120 | 100310786 | 100307122 | 100307122 |
| 7 | 100307123 | 100307122 | 10301 | 100307123 | 100307123 |
| 8 | 100312951 | 100307123 | 1050 | 100312951 | 100312951 |
| 9 | 10058 | 10058 | 10962 | 100859930 | 100859930 |
| 10 | 100859930 | 10669 | 1387 | 10286 | 10286 |
| 11 | 10286 | 10752 | 154215 | 11170 | 11170 |
| 12 | 10669 | 11082 | 1728 | 11178 | 11178 |
| 13 | 10752 | 11170 | 1788 | 11236 | 11236 |
| 14 | 11082 | 11178 | 2064 | 114568 | 114568 |
| 15 | 11170 | 11235 | 2066 | 1499 | 1499 |
| 16 | 11178 | 11236 | 2078 | 1630 | 1630 |
| 17 | 11235 | 116449 | 2120 | 201163 | 201163 |
| 18 | 114568 | 117289 | 219285 | 22800 | 22800 |
| 19 | 116449 | 117583 | 220107 | 22861 | 22861 |
| 20 | 117289 | 125 | 23048 | 23373 | 23373 |
| 21 | 117583 | 1359 | 23092 | 256646 | 256646 |
| 22 | 125 | 14 | 2322 | 257641 | 257641 |
| 23 | 1359 | 140947 | 23305 | 283600 | 283600 |
| 24 | 14 | 154215 | 23365 | 283902 | 283902 |
| 25 | 140947 | 157313 | 25 | 284654 | 284654 |
| 26 | 1499 | 1670 | 2521 | 3265 | 3265 |
| 27 | 154215 | 1671 | 2623 | 344967 | 344967 |
| 28 | 157313 | 171558 | 2624 | 3482 | 3482 |
| 29 | 1670 | 1879 | 26511 | 355 | 355 |
| 30 | 1671 | 1894 | 27004 | 3621 | 3621 |
| 31 | 171558 | 1956 | 2782 | 378825 | 378825 |
| 32 | 1879 | 1981 | 28982 | 3845 | 3845 |
| 33 | 1894 | 200958 | 30012 | 4233 | 4233 |
| 34 | 1956 | 201633 | 3131 | 4437 | 4437 |
| 35 | 1981 | 214 | 3195 | 4486 | 4486 |
| 36 | 200958 | 221935 | 3196 | 4893 | 4893 |
| 37 | 201633 | 2271 | 3205 | 4968 | 4968 |
| 38 | 214 | 22858 | 3344 | 50975 | 50975 |
| 39 | 221935 | 23418 | 338436 | 51079 | 51079 |
| 40 | 2271 | 23513 | 3627 | 51741 | 5157 |
| 41 | 22858 | 246184 | 3659 | 5290 | 51741 |
| 42 | 23418 | 2532 | 3717 | 55193 | 5290 |
| 43 | 2532 | 253559 | 3815 | 5546 | 55193 |
| 44 | 253559 | 260436 | 3845 | 55653 | 5546 |
| 45 | 257641 | 26230 | 3932 | 5727 | 55653 |
| 46 | 260436 | 2649 | 3976 | 5921 | 5727 |
| 47 | 26230 | 2668 | 3977 | 5932 | 5921 |
| 48 | 2668 | 27074 | 4004 | 5979 | 5932 |
| 49 | 27074 | 27152 | 4005 | 6049 | 5979 |
| 50 | 27152 | 284654 | 4026 | 6317 | 6049 |
| 51 | 284654 | 285852 | 4066 | 6318 | 6317 |
| 52 | 285852 | 285955 | 4170 | 6608 | 6318 |
| 53 | 285955 | 286204 | 4291 | 6762 | 6608 |
| 54 | 28636 | 286205 | 4300 | 6927 | 6762 |
| 55 | 28637 | 28636 | 4302 | 6928 | 6927 |
| 56 | 28638 | 28637 | 4309 | 7030 | 6928 |
| 57 | 28639 | 28638 | 4311 | 7157 | 7030 |
| 58 | 30012 | 28639 | 4343 | 7253 | 7157 |
| 59 | 3059 | 30012 | 4602 | 728655 | 7253 |
| 60 | 3101 | 3059 | 4629 | 729582 | 728655 |
| 61 | 3196 | 3101 | 4683 | 7428 | 729582 |
| 62 | 3344 | 3196 | 472 | 79052 | 7428 |
| 63 | 3382 | 3344 | 4763 | 79577 | 79052 |
| 64 | 338436 | 3382 | 4869 | 8066 | 79577 |
| 65 | 340205 | 338436 | 4926 | 8312 | 8066 |
| 66 | 340206 | 340205 | 4928 | 841 | 8312 |
| 67 | 344967 | 340206 | 5079 | 84441 | 841 |
| 68 | 3482 | 3575 | 5087 | 84925 | 84441 |
| 69 | 3575 | 3592 | 5089 | 8537 | 84925 |
| 70 | 3592 | 3593 | 5090 | 8643 | 8537 |
| 71 | 3593 | 3659 | 5371 | 8795 | 8643 |
| 72 | 3659 | 3702 | 54809 | 9562 | 8795 |
| 73 | 3702 | 3815 | 55640 | 999 | 9562 |
| 74 | 3815 | 3905 | 5713 |  | 999 |
| 75 | 3905 | 394263 | 57213 |  |  |
| 76 | 394263 | 3950 | 574016 |  |  |
| 77 | 3950 | 3973 | 574028 |  |  |
| 78 | 3973 | 4117 | 57591 |  |  |
| 79 | 4117 | 4170 | 5781 |  |  |
| 80 | 4170 | 4233 | 581 |  |  |
| 81 | 4233 | 4332 | 5910 |  |  |
| 82 | 4332 | 440590 | 5914 |  |  |
| 83 | 440590 | 440712 | 596 |  |  |
| 84 | 440712 | 441272 | 602 |  |  |
| 85 | 441272 | 441273 | 613 |  |  |
| 86 | 441273 | 445347 | 6418 |  |  |
| 87 | 4437 | 4582 | 6455 |  |  |
| 88 | 445347 | 4602 | 64783 |  |  |
| 89 | 4486 | 4739 | 6574 |  |  |
| 90 | 4582 | 4819 | 6739 |  |  |
| 91 | 4602 | 4846 | 6777 |  |  |
| 92 | 4739 | 4897 | 6886 |  |  |
| 93 | 4819 | 4968 | 6887 |  |  |
| 94 | 4846 | 5079 | 6946 |  |  |
| 95 | 4893 | 50852 | 6988 |  |  |
| 96 | 4897 | 5087 | 7015 |  |  |
| 97 | 4968 | 5089 | 7068 |  |  |
| 98 | 50852 | 51057 | 7704 |  |  |
| 99 | 5087 | 51072 | 7913 |  |  |
| 100 | 5089 | 51095 | 79870 |  |  |
| 101 | 51057 | 51150 | 8009 |  |  |
| 102 | 51072 | 51237 | 8021 |  |  |
| 103 | 51095 | 5133 | 8028 |  |  |
| 104 | 51150 | 5134 | 80714 |  |  |
| 105 | 51237 | 51362 | 8079 |  |  |
| 106 | 5133 | 51696 | 8101 |  |  |
| 107 | 5134 | 5290 | 8115 |  |  |
| 108 | 51362 | 53335 | 8178 |  |  |
| 109 | 51696 | 54209 | 8201 |  |  |
| 110 | 5290 | 54210 | 8205 |  |  |
| 111 | 53335 | 55024 | 8301 |  |  |
| 112 | 54209 | 55143 | 861 |  |  |
| 113 | 54210 | 55193 | 865 |  |  |
| 114 | 55024 | 5546 | 867 |  |  |
| 115 | 55143 | 55536 | 8847 |  |  |
| 116 | 55193 | 55665 | 8915 |  |  |
| 117 | 5546 | 56667 | 9623 |  |  |
| 118 | 55536 | 56941 |  |  |  |
| 119 | 55665 | 57216 |  |  |  |
| 120 | 56667 | 5727 |  |  |  |
| 121 | 56941 | 57650 |  |  |  |
| 122 | 57216 | 57805 |  |  |  |
| 123 | 57650 | 57863 |  |  |  |
| 124 | 57805 | 5788 |  |  |  |
| 125 | 57863 | 5798 |  |  |  |
| 126 | 5788 | 5910 |  |  |  |
| 127 | 5798 | 5921 |  |  |  |
| 128 | 5910 | 607 |  |  |  |
| 129 | 5921 | 6383 |  |  |  |
| 130 | 607 | 63924 |  |  |  |
| 131 | 63924 | 641700 |  |  |  |
| 132 | 641700 | 64395 |  |  |  |
| 133 | 64395 | 64396 |  |  |  |
| 134 | 64396 | 64641 |  |  |  |
| 135 | 64641 | 6608 |  |  |  |
| 136 | 6608 | 673 |  |  |  |
| 137 | 673 | 683 |  |  |  |
| 138 | 683 | 6886 |  |  |  |
| 139 | 6886 | 6887 |  |  |  |
| 140 | 6932 | 6932 |  |  |  |
| 141 | 6946 | 6946 |  |  |  |
| 142 | 6966 | 6966 |  |  |  |
| 143 | 6967 | 6967 |  |  |  |
| 144 | 6988 | 6988 |  |  |  |
| 145 | 728655 | 7412 |  |  |  |
| 146 | 729582 | 7428 |  |  |  |
| 147 | 7412 | 7462 |  |  |  |
| 148 | 7428 | 7851 |  |  |  |
| 149 | 7462 | 7864 |  |  |  |
| 150 | 7851 | 78996 |  |  |  |
| 151 | 7864 | 790952 |  |  |  |
| 152 | 78996 | 7956 |  |  |  |
| 153 | 79052 | 79577 |  |  |  |
| 154 | 790952 | 7962 |  |  |  |
| 155 | 7956 | 7963 |  |  |  |
| 156 | 79577 | 79679 |  |  |  |
| 157 | 7962 | 79699 |  |  |  |
| 158 | 7963 | 79865 |  |  |  |
| 159 | 79679 | 8000 |  |  |  |
| 160 | 79699 | 80157 |  |  |  |
| 161 | 79865 | 80177 |  |  |  |
| 162 | 80157 | 80380 |  |  |  |
| 163 | 80177 | 8317 |  |  |  |
| 164 | 8317 | 835 |  |  |  |
| 165 | 835 | 83879 |  |  |  |
| 166 | 83879 | 84141 |  |  |  |
| 167 | 841 | 84433 |  |  |  |
| 168 | 84141 | 84824 |  |  |  |
| 169 | 84433 | 84868 |  |  |  |
| 170 | 84824 | 84925 |  |  |  |
| 171 | 84868 | 8521 |  |  |  |
| 172 | 84925 | 8555 |  |  |  |
| 173 | 8521 | 8556 |  |  |  |
| 174 | 8556 | 8643 |  |  |  |
| 175 | 8643 | 8697 |  |  |  |
| 176 | 8697 | 8795 |  |  |  |
| 177 | 8795 | 8820 |  |  |  |
| 178 | 8820 | 8915 |  |  |  |
| 179 | 8915 | 9125 |  |  |  |
| 180 | 9125 | 91272 |  |  |  |
| 181 | 91272 | 914 |  |  |  |
| 182 | 914 | 91937 |  |  |  |
| 183 | 91937 | 9247 |  |  |  |
| 184 | 9247 | 9275 |  |  |  |
| 185 | 9275 | 962 |  |  |  |
| 186 | 962 | 9750 |  |  |  |
| 187 | 9750 | 9812 |  |  |  |
| 188 | 9812 | 9844 |  |  |  |
| 189 | 9844 | 988 |  |  |  |
| 190 | 988 | 991 |  |  |  |
| 191 | 991 | 993 |  |  |  |
| 192 | 993 | 995 |  |  |  |
| 193 | 995 | 998 |  |  |  |
| 194 | 998 |  |  |  |  |
